# Acarbose differentially affects gut microbial metabolism of maltose and sucrose, distinctly modulating host metabolism

**DOI:** 10.1016/j.isci.2026.117889

**Published:** 2026-10-13

**Authors:** Hayato Yoshikawa, Eunyoung Lee, Ryo Hatano, Yuki Taki, Akiko Takaya, Akifumi Eguchi, Yoshiyuki Goto, Junki Miyamoto, Ikuo Kimura, Tomoaki Tanaka, Takashi Miki

**Affiliations:** 1 Depertment of Medical Physiology, Graduate School of Medicine, Chiba University, Chiba 260-8670, Japan; 2 Institute for Advanced Academic Research, Chiba University, Chiba 260-8522, Japan; 3 Reserch Institute of Disaster Medicine (RIDM), Chiba University, Chiba 260-0856, Japan; 4 Department of Molecular Diagnosis, Graduate School of Medicine, Chiba University, Chiba 260-8670, Japan; 5 Division of Molecular Immunology, Medical Mycology Research Center, Chiba University, Chiba 260-8673, Japan; 6 Department of Infection Control Science, Graduate School of Pharmaceutical Sciences, Chiba University, Chiba 260-8675, Japan; 7 Department of Sustainable Health Science, Center for Preventive Medical Sciences, Chiba University, Chiba 263-8522, Japan; 8 Department of Applied Biological Science, Graduate School of Agriculture, Tokyo University of Agriculture and Technology, Tokyo 183-8509, Japan; 9 Laboratory of Molecular Metabolism, Graduate School of Biostudies, Kyoto University, Kyoto 606-8501, Japan

**Keywords:** acarbose, α-GI, gut microbiota, SCFA, incretin, obesity

## Abstract

Gut microbiota act as beneficial symbionts of the hosts. Administration of α-glucosidase inhibitors (α-GIs) is considered to promote the microbiota’s carbohydrate availability, modulating short-chain fatty acid (SCFA) production. However, it remains unclear whether host-microbiota interaction differs depending on α-GI types (e.g., acarbose versus miglitol) and carbohydrate types co-administered. In this study, we demonstrate that acarbose is transported through bacterial outer membrane porins, LamB and ScrY, and inhibits maltose metabolism in *Escherichia coli*. Furthermore, studies using *Klebsiella pneumoniae* reveal that acarbose suppresses utilization of maltose, but not that of sucrose. When co-administered with acarbose in mice, sucrose, relative to maltose, enhances SCFA production, suppresses glucose-dependent insulinotropic polypeptide (GIP) secretion, and protects against high-fat diet-induced obesity, underscoring the importance of sucrose as a microbiota-dependent beneficial factor, but not a culprit in host metabolism. In conclusion, acarbose regulates host metabolic processes partly by modulating gut microbial metabolism in a carbohydrate-type-dependent manner.

## Highlights

• Acarbose is transported by bacterial transporters to inhibit maltose metabolism
• With acarbose, sucrose, but not maltose, increases SCFA and modulates incretins
• Acarbose increases microbiota involved in SCFA production under an HFD+sucrose diet
• Acarbose more effectively protects against obesity with sucrose than with maltose

## Graphical abstract

**Figure:**
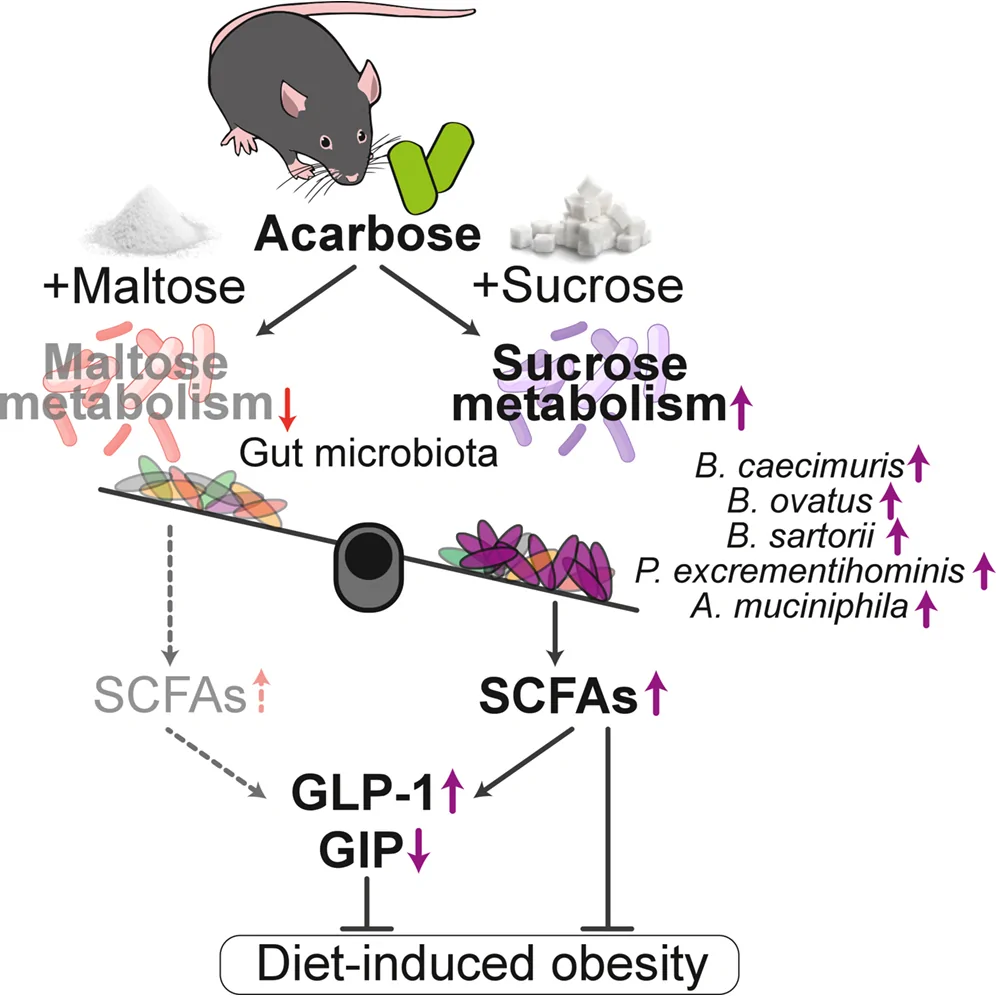

## Teaser

Biomolecules; Microbial metabolism; Metabolic flux analysis

## Introduction

Mammals, including humans and mice, harbor approximately 10^14^ microorganisms within the intestinal lumen, an environment characterized by a stable temperature, anaerobic conditions, and abundant nutrients.^1^ Survival and proliferation of the gut microbiota depend primarily on host diet-derived nutrients. In turn, the host benefits from the gut microbiota in various aspects, thereby rendering the gut microbiota essential metabolic partners rather than passive inhabitants.^2^^,^^3^^,^^4^ The importance of maintaining a normal gut microbiome is underscored by numerous studies linking alterations in microbial composition, termed dysbiosis, to a wide range of diseases.^5^^,^^6^ Dysbiosis can be induced by various factors, including oral antibiotic administration and a Western-style high-fat diet. Whereas the former can cause pseudomembranous colitis mediated by expansion of *Clostridioides difficile*, the latter is associated with metabolic disorders, including obesity and type 2 diabetes.^7^^,^^8^

Among the factors produced by the microbiota, short-chain fatty acids (SCFAs) derived from carbohydrate fermentation are critical signaling molecules.^9^^,^^10^ SCFAs suppress adiposity^11^ and modulate enteroendocrine signaling by regulating the secretion of incretin hormones, including glucagon-like peptide-1 (GLP-1) and glucose-dependent insulinotropic polypeptide (GIP).^12^^,^^13^^,^^14^ These incretins potentiate insulin secretion from pancreatic β cells and contribute to energy homeostasis. Notably, SCFAs have been reported to increase GLP-1 secretion but reduce GIP secretion, indicating their coordinated yet distinct control of incretin responses.^12^^,^^14^

Mammals ingest most dietary carbohydrates in the form of polysaccharides, which are hydrolyzed into monosaccharides by intestinal α-glucosidases before absorption. By exploiting this property, α-glucosidase inhibitors (α-GIs) have been developed to mitigate postprandial hyperglycemia.^15^ In the presence of α-GIs, undigested polysaccharides remain in the gut lumen and function as microbiota-accessible carbohydrates (MACs).^14^

We previously reported that co-administration of the α-GI miglitol with maltose enhances microbiota-derived SCFA production, accompanied by increased GLP-1 secretion and suppressed GIP secretion.^14^^,^^16^ Because these responses are entirely abolished in germ-free mice,^14^ microbiota-derived factors, including SCFAs, appear to be essential mediators of incretin secretion in response to increased MAC availability. However, an intriguing discrepancy exists: acarbose, despite belonging to the same pharmacological class, exhibits significantly weaker effects on incretin secretion than miglitol.^14^^,^^16^ The mechanisms by which these two drugs differentially influence microbiota-mediated maltose metabolism remain unclear.

Here, using complementary *in vivo* and *in vitro* approaches, we demonstrate that acarbose, but not miglitol, modulates gut microbial carbohydrate metabolism by selectively inhibiting bacterial maltose utilization without affecting sucrose metabolism. Consequently, a single bolus co-administration of sucrose with acarbose enhances SCFA production and significantly modulates incretin secretion in mice. Under high-fat diet conditions, chronic acarbose supplementation with high sucrose—but not high maltose—protects against diet-induced obesity (DIO) concomitant with elevated plasma propionate and butyrate concentrations and suppressed GIP secretion. Together, these findings reveal that sucrose, a dietary component traditionally considered a metabolic risk factor, can instead confer protection against obesity when co-administered with acarbose by functioning as an anti-obesity prebiotic.

## Results

### Miglitol, but not acarbose, enhances maltose-derived SCFA production in mice

To determine whether distinct α-GIs differentially affect postprandial SCFA production, the mice were subjected to an oral maltose challenge with or without co-administration of either miglitol or acarbose (Figure 1A). Based on a preliminary oral maltose tolerance test, doses of 10 mg/kg miglitol and 40 mg/kg acarbose were selected because they produced comparable suppression of postprandial glycemic responses (Figure S1A). While miglitol and acarbose significantly attenuated postprandial blood glucose elevations compared with maltose alone (Figure 1B), they exerted markedly different effects on SCFA production. When co-administered with maltose, portal plasma concentrations of all SCFAs except *iso*-valerate were significantly increased by miglitol, but not by acarbose (Figure 1C). These results suggest that, despite a presumed increase in luminal maltose availability following α-GI co-administration, acarbose, but not miglitol, may limit microbial maltose utilization.

**Figure 1:**
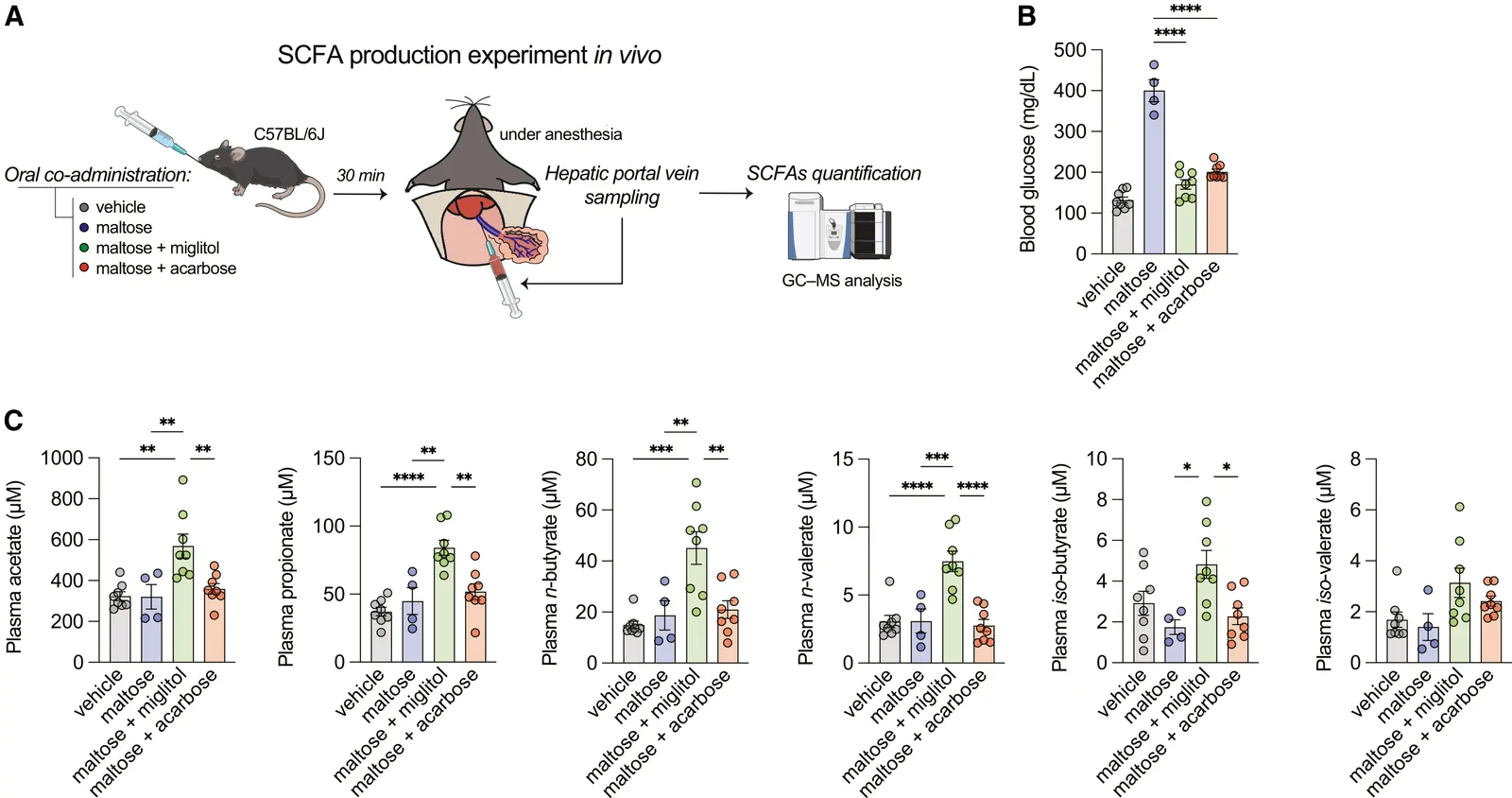
Miglitol, but not acarbose, enhances maltose-derived SCFA production in mice (A) Schematic illustration of the *in vivo* SCFA production measurement. (B and C) Blood glucose levels (B) and plasma SCFA concentrations of the portal vein (C) at 30 min after oral administration of maltose alone, maltose + miglitol, or maltose + acarbose (*n* = 4–8 biological replicates). Vehicle-treated mice served as controls. In (B), data for the vehicle and maltose + miglitol groups are reproduced from Ma et al. (2022).^17^ These groups were part of the same experiment from which the data newly presented in this study were derived. Data are presented as mean ± SEM. ∗*p* < 0.05, ∗∗*p* < 0.01, ∗∗∗*p* < 0.001, and ∗∗∗∗*p* < 0.0001, as determined by one-way ANOVA followed by Tukey’s multiple-comparisons test. See also Figure S1.

### Acarbose uptake through the maltoporin LamB inhibits maltose metabolism in *Escherichia coli*

To compare the effects of acarbose and miglitol on bacterial maltose metabolism *in vitro*, we used *Escherichia coli* (*E. coli*) as a genetically tractable proof-of-concept model harboring the well-characterized maltose transport and metabolic system (Table 1). When cultured in M9 minimal medium containing maltose as the sole carbon source (M9/maltose), *E. coli* grew and released acetate (Figure 2A). Addition of miglitol did not affect bacterial growth or acetate production, whereas acarbose markedly suppressed both (Figure 2A). However, when the carbon source in the medium was changed from maltose to glucose, acarbose failed to suppress bacterial growth; rather, it promoted the growth in the late phase in M9/glucose medium (Figure 2B). In contrast, the addition of miglitol did not affect bacterial growth (Figure 2B). To clarify whether the growth inhibitory effect of acarbose requires its incorporation into bacteria, we generated *E. coliΔlamB* strain lacking the outer membrane maltoporin LamB. In the wild-type *E. coli*, LamB protein expression was higher under M9/maltose medium than under M9/glucose medium (Figure 2C) in accord with the notion that LamB expression is regulated by the maltose operon.^18^ In the *ΔlamB* strain, LamB expression was absent, while its expression was restored by its constitutive genetic rescue with the plasmid (*ΔlamB/lamB*_*Ec*_) to a level comparable to that in wild-type *E. coli* under M9/maltose medium (Figure 2C). In the *ΔlamB* strain, the growth inhibition by acarbose was attenuated (Figure 2D), while its genetic rescue restored the acarbose-induced growth inhibition (Figure 2E), indicating that LamB-mediated acarbose uptake suppresses its growth on maltose. As described previously,^18^ LamB expression was suppressed in M9/glucose medium (Figure 2C). Therefore, no growth suppression by acarbose in wild-type *E. coli* cultured in M9/glucose (Figure 1B) may be attributable to the reduced LamB expression. To rule out this possibility, we examined the bacterial growth of *ΔlamB/lamB*_*Ec*_ strain that possesses constitutive LamB expression even in M9/glucose medium (Figure 2C). Growth of the *ΔlamB/lamB*_*Ec*_ strain was not suppressed by acarbose in M9/glucose medium (M9/glucose + acarbose) (Figure 2F), showing that the presence of acarbose inside the cells does not affect bacterial growth on glucose. Given its structural similarity with maltotetraose (Figure 2G), acarbose is presumed to be transported into cytoplasm via the inner membrane ABC transporter MalEFGK_2_, comprising *malE*, *malF*, *malG*, and *malK* (Figure 2H).^19^ These genes are transactivated by the transcription factor MalT, which is separated from MalEFGK_2_–MalT complex when MalEFGK_2_ undergoes morphological changes during substrate transport (Figure 2H).^19^ Therefore, we sought to evaluate the transport of acarbose into the *E. coli* cytosol by monitoring the transactivation of *malE*, *malF*, and *malK* upon addition of acarbose to M9 medium. Quantitative RT-PCR analysis revealed that acarbose alone increased the expression of *malE*, *malF*, and *malK* in a dose-dependent manner (Figure 2I), suggesting that acarbose mimics maltotetraose and activates the maltose regulon. To determine whether growth inhibition was attributable to the valienamine moiety of acarbose, which contributes to its α-glucosidase inhibitory activity (Figure S2A), or competition with the bacterial maltose utilization system, we examined the effects of another α-GI, voglibose (Figure S2B), which contains a valiolamine moiety (Figure S2C), and maltotetraose, which has a structure and molecular size similar to those of acarbose but lacks α-GI activity, on the growth of *E. coli*. Neither voglibose or maltotetraose inhibited the growth of *E. coli* in M9/maltose medium at 2 mM (Figures S2D and S2E). The effect of acarbose on maltose metabolism in *E. coli* was assessed by metabolome analysis. When maltose was added to M9 medium, pyruvate and its downstream glycolytic metabolites (acetyl-CoA, succinic acid, and lactic acid) increased, whereas upstream metabolites (phosphoenolpyruvate and 3-phosphoglycerate) decreased, indicating that maltose metabolism was upregulated (Figure 2J). By contrast, the further addition of acarbose to M9/maltose medium resulted in intracellular accumulation of both undigested acarbose and maltose, whereas acarbose significantly decreased pyruvate and other downstream metabolites of glycolysis (Figure 2J).

**Table 1:** Bacterial strains used in this study

Strain	Description	Source
*E. coli* DH5α	F-, Φ80d*lacZ*ΔM15, Δ(*lacZYA-argF*)U169, *deoR*, *recA1*, *endA1*, *hsdR*17(rK-, mK+), *phoA*, *supE*44, *λ*-, *thi*-1, *gyrA*96, *relA*1	Our collection
*E. coli* W3110	F-, *λ*-, *rphI*	Our collection
*K. pneumoniae* Kpc01	bloodstream clinical isolate	Yamanaka et al.^28^
*E. coli* W3110 Δ*lamB*	Δ*lamB*::FRT in *E. coli* W3110	This study
*E. coli* W3110 Δ*lamB*/p*scrY*	*E. coli* W3110 harboring p*scrY*	This study
*E. coli* W3110 Δ*lamB*/p*lamB*_*Ec*_	*E. coli* W3110 harboring p*lamB*_*Ec*_	This study
*E. coli* W3110 Δ*lamB*/p*lamB*_*Kp*_	*E. coli* W3110 harboring p*lamB*_*Kp*_	This study

**Figure 2:**
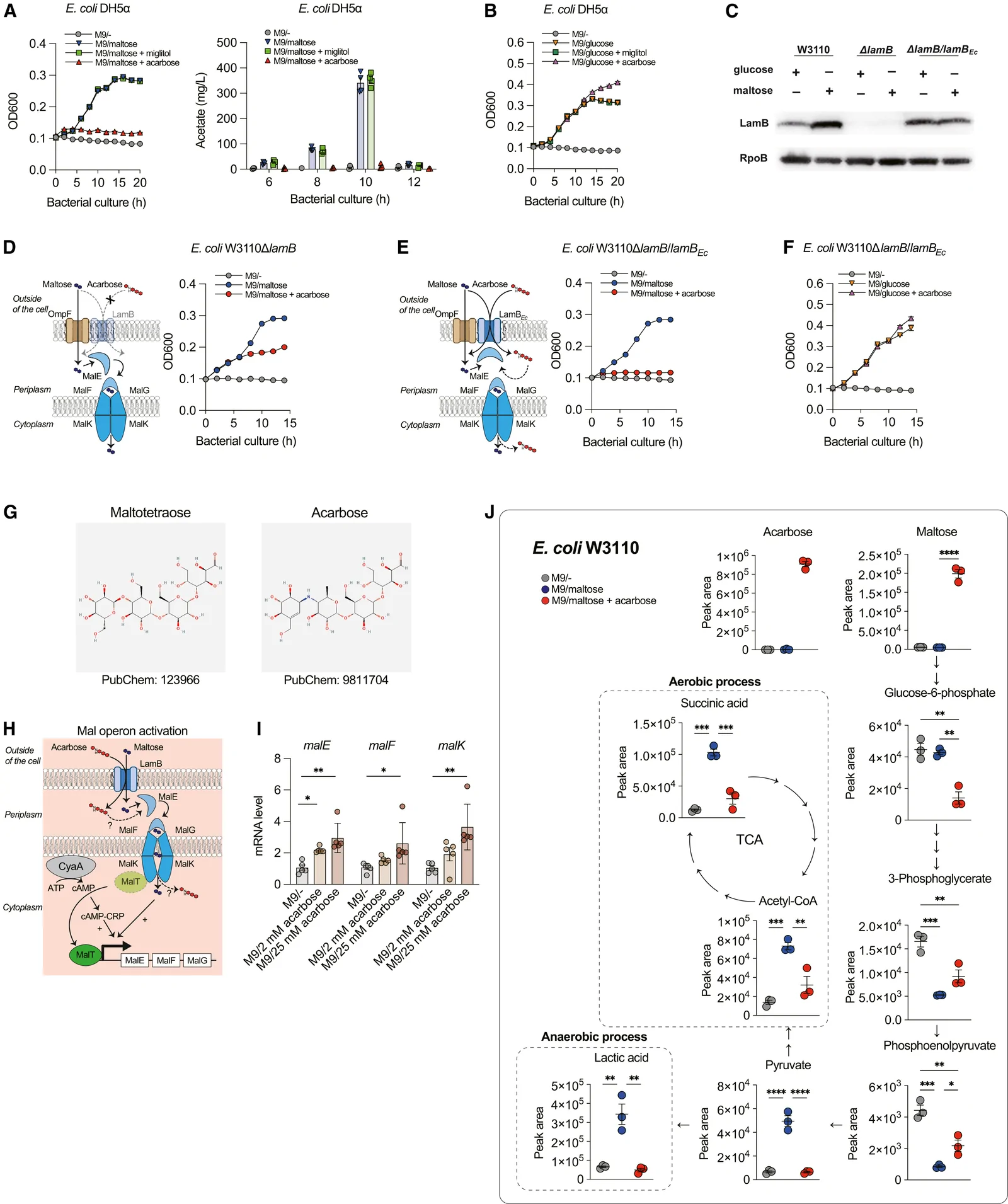
Acarbose uptake through the maltoporin LamB inhibits maltose metabolism in *Escherichia coli* (A and B) Bacterial growth curves of *E. coli* (A and B) and acetate released into the medium (A) (*n* = 4 biological replicates). (C) Immunoblot analysis of LamB and the RNA polymerase subunit beta (RpoB) of three *E. coli* strains harvested after 6 h of culture (*n* = 1 biological sample, independent experiment). (D–F) Bacterial growth curves of genetically modified *E. coli* strains (*n* = 4 biological replicates). (G) Structural comparison of maltotetraose (PubChem 123966) and acarbose (PubChem 9811704). (H) Schematic illustration of maltodextrin transport and mal operon activation of *E. coli*. (I) RT-qPCR analysis of *malE*, *malF*, and *malK* of *E. coli* (*n* = 5 biological replicates). (J) Intracellular metabolite profiles of *E. coli* W3110 treated in M9 medium supplemented with maltose, with or without acarbose (*n* = 3 independent biological samples in each group). Data are presented as mean ± SEM. ∗*p* < 0.05, ∗∗*p* < 0.01, ∗∗∗*p* < 0.001, and ∗∗∗∗*p* < 0.0001, as determined by one-way ANOVA followed by Tukey’s multiple-comparisons test. See also Figure S2.

### Computational simulations predict that acarbose interacts with the bacterial enzymes MalQ and MalP

In *E. coli*, maltose metabolism is primarily mediated by the amylomaltase MalQ and the maltodextrin phosphorylase MalP, which cooperatively drive cytosolic glycolysis.^18^ Cytosolic maltose is converted by MalQ into maltodextrins with varying degrees of polymerization, whereas MalP catalyzes phosphorolysis from the non-reducing ends of maltodextrins, with maltopentaose reported as the shortest substrate,^18^ thereby releasing glucose-1-phosphate. Given that acarbose-like molecules are known to bind the catalytic site of MalQ,^20^ the observed intracellular accumulation of undigested maltose upon acarbose supplementation (Figure 2J) supports functional inhibition of MalQ activity *in vivo* in *E. coli*. To evaluate this possibility, we performed *in silico* docking simulations using the substrate-bound conformation of MalQ.^20^ Binding affinities at the catalytic sites (Asp448, Glu496, and Asp548) increase with the degree of polymerization (maltose < maltotriose < maltotetraose), while acarbose exhibits an affinity comparable to maltotriose (Figure 3A). Consistent with this trend, docking analysis predicted that maltose forms a hydrogen bond only with Asp548 (putative transition state stabilizer; 2.1 Å), whereas acarbose additionally engages Glu496 (acid/base catalyst; 2.6 Å). In contrast, maltotetraose is predicted to interact with all three catalytic residues, including nucleophilic side chain Asp448 (Figure 3A). To assess whether differential affinity in these interactions is reflected in the bacterial growth, *E. coli* was cultured in M9 minimum medium containing either maltose, maltotriose, maltotetraose, or maltohexaose, with or without acarbose. Acarbose completely suppressed *E. coli* growth on maltose throughout the observation period (48 h), whereas growth resumed after 24 h in M9 medium containing either maltotriose, maltotetraose, or maltohexaose (Figure 3B), indicating that the inhibitory effect of acarbose depends on α-1,4-glucan chain length. We also performed docking simulations targeting the cytosolic maltodextrin phosphorylase MalP. Acarbose was predicted to bind more favorably to the catalytic site than maltooligosaccharides, adopting a pose closer to aromatic residues,^21^ including Tyr280 (subsite +2) and Tyr613 (subsite +4) (Figure 3C). Consistently, metabolomic analysis revealed intracellular accumulation of longer maltodextrins, including maltotriose, maltotetraose, maltopentaose, and maltohexaose, when the cells were treated with acarbose (M9/maltose + acarbose), indicating that acarbose impairs maltodextrin phosphorolysis by MalP (Figure 3D). Although exogenous supply of longer maltooligosaccharides supported bacterial growth even in the presence of acarbose (Figure 3B), intracellular accumulation of these substrates failed to restore the glycolytic process (Figures 2J and 3D), suggesting that maltose is not efficiently converted into longer maltooligosaccharides by MalQ, thereby limiting subsequent phosphorolysis by MalP. Together, these findings suggest that LamB in *E. coli* mediates the uptake of acarbose, which is subsequently transported into the cytoplasm by MalEFGK_2_, where acarbose may interfere with both MalQ- and MalP-mediated cytosolic maltodextrin metabolism, leading to suppressed glycolytic flux and impaired growth.

**Figure 3:**
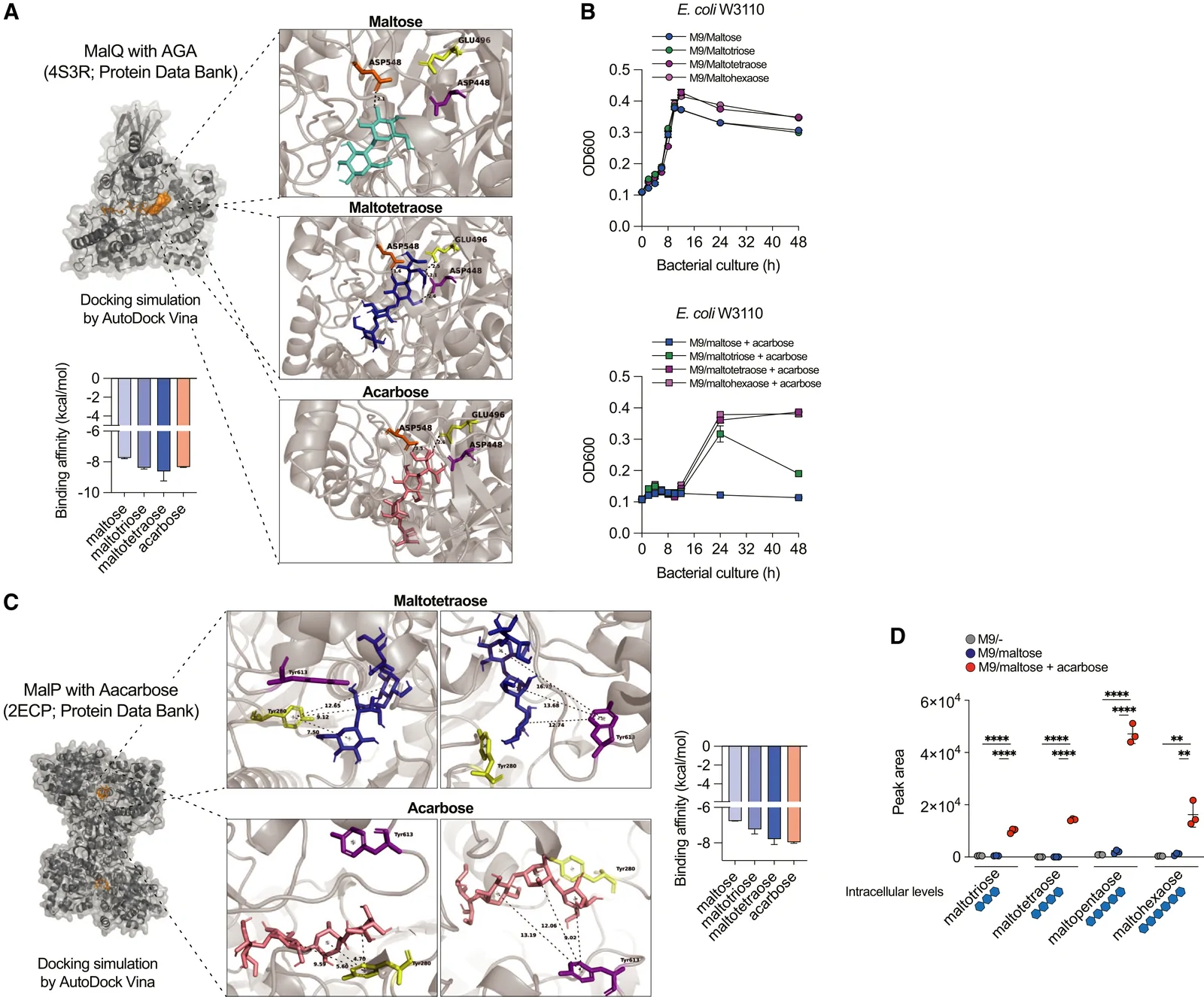
Computational simulations predict that acarbose interacts with the bacterial enzymes MalQ and MalP (A) Docking simulations using AutoDock Vina predicted the binding of maltooligosaccharides and acarbose within the catalytic pocket of MalQ (MalQ from *E. coli* in complex with acarviosine-glucose-acarbose (AGA); PDB 4S3R), with estimated binding energies (kcal mol^−1^) (maltose −7.74 ± 0.05; maltotriose −8.40 ± 0.09; maltotetraose −8.61 ± 0.62; acarbose −8.32 ± 0.03; *n* = 5 independent docking simulations). (B) Growth curves of *E. coli* cultured in M9/maltose, M9/maltotriose, M9/maltotetraose, and M9/maltohexaose in the presence or absence of acarbose (*n* = 3 biological replicates). (C) Docking simulations predicted the binding of maltooligosaccharides and acarbose within the catalytic pocket of MalP (MalP form *E. coli* in complex with acarbose; PDB 2ECP), with estimated binding energies (kcal mol^−1^) (maltose −6.76 ± 0.01; maltotriose −7.23 ± 0.26; maltotetraose −7.79 ± 0.30; acarbose −7.97 ± 0.06; *n* = 5 independent docking simulations). (D) Intracellular levels of maltooligosaccharides under the same conditions as in Figure 2J (*n* = 3 biological replicates). Colors indicate ligands used for docking: light blue, maltose; blue, maltotriose; navy blue, maltotetraose; pink, acarbose. Data are presented as mean ± SD in A and C, and as mean ± SEM in B and D. ∗*p* < 0.05, ∗∗*p* < 0.01, ∗∗∗*p* < 0.001, and ∗∗∗∗*p* < 0.0001, as determined by one-way ANOVA followed by Tukey’s multiple-comparisons test.

### Acarbose is transported by the sucrose porin ScrY and suppresses bacterial growth on maltose but not on sucrose in *Klebsiella pneumoniae*

LamB belongs to a family of carbohydrate-specific porin.^22^ This family also includes ScrY, the sucrose-preferring member identified in plasmid pUR400 involved in sucrose metabolism in *Klebsiella pneumoniae* (*K. pneumoniae*).^23^ Although ScrY shares high structural and functional similarity with LamB,^24^^,^^25^ it remains unknown whether ScrY recognizes and transports acarbose. We evaluated this by testing whether genetic transduction of ScrY in LamB-deficient *E. coli* (*ΔlamB/scrY*) restores growth inhibition by acarbose. In *ΔlamB/scrY* strain, acarbose inhibited growth on maltose similarly to that observed in wild-type *E. coli* W3110 (Figure 4A), indicating that ScrY transports acarbose as well as LamB. In addition, transduction of LamB from *K. pneumoniae* to the *E. coliΔlamB* strain (*ΔlamB/lamB*_*Kp*_) also restored the growth inhibition by acarbose when grown on maltose (Figure 4B). Given that acarbose is transported by ScrY and LamB of *K. pneumoniae*, we next examined whether acarbose inhibits growth of *K. pneumoniae*, which utilizes maltose and sucrose as carbon sources. As reported,^26^^,^^27^ *K. pneumoniae* grew in both M9/maltose and M9/sucrose media. Acarbose inhibited the growth of *K. pneumoniae* on maltose not by completely arresting growth but by delaying it. By contrast, when grown on sucrose, acarbose failed to suppress its growth but rather enhanced it (Figure 4C). Consistent with its growth pattern, acarbose delayed acetate accumulation when *K. pneumoniae* was cultured on maltose, abolishing acetate production at 4 h, whereas acetate levels increased at 8 and 10 h (Figure 4D). By contrast, acarbose did not substantially affect acetate accumulation when cultured on sucrose (Figure 4D). Collectively, these *in vitro* analyses indicate that acarbose affects bacterial metabolism differently, depending on the bacterial species and the types of carbohydrates available.

**Figure 4:**
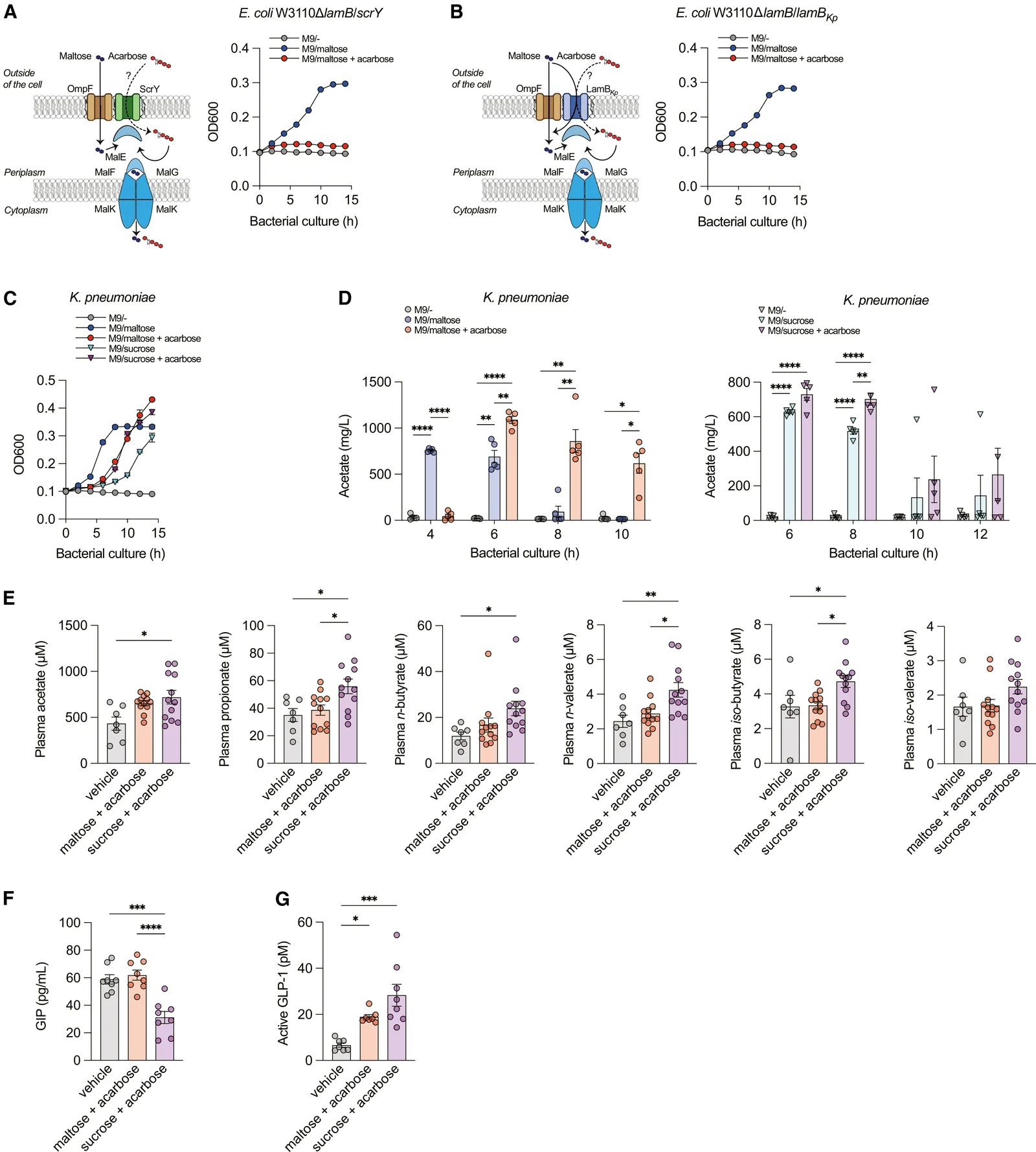
Acarbose is transported by the sucrose porin ScrY and decreases GIP while increasing GLP-1 when co-administered with sucrose in mice (A and B) Bacterial growth curves of *E. coli*Δ*lamB* strain complemented with *K. pneumoniae*-derived ScrY (Δ*lamB*/*scrY*) (A) and LamB (Δ*lamB*/*lamB*_*Kp*_) (B) (*n* = 4 biological replicates). (C) Growth curves of *K. pneumoniae* cultured in M9/maltose or M9/sucrose in the presence or absence of acarbose (*n* = 3 biological replicates). (D) Acetate concentrations in the culture medium of *K. pneumoniae* under the conditions shown in (C) (*n* = 5 biological replicates). *p* values from an unpaired *t* test with Welch’s correction: ∗*p* < 0.05. (E–G) Portal SCFA (E), GIP (F), and GLP-1 (G) levels at 30 min after oral co-administration of acarbose with maltose (maltose + acarbose) or sucrose (sucrose + acarbose) (*n* = 7–12 biological replicates). Data are presented as mean ± SEM. ∗*p* < 0.05, ∗∗*p* < 0.01, ∗∗∗*p* < 0.001, and ∗∗∗∗*p* < 0.0001, as determined by one-way ANOVA followed by Tukey’s multiple-comparisons test.

### When co-administered with acarbose in mice, sucrose more potently increases portal SCFAs, decreases GIP, and increases GLP-1 than maltose

As shown above, acarbose inhibited bacterial growth and acetate production in *K. pneumoniae* when cultured on maltose, but not on sucrose. Because *K. pneumoniae* is not a typical commensal bacterium but an opportunistic pathobiont in the human gut,^28^ the response to acarbose observed in this strain alone may not reflect the response of commensal gut bacteria involved in sucrose metabolism. Therefore, we next assessed the effect of acarbose on plasma SCFA levels in healthy mice after its co-administration with maltose or sucrose. Portal SCFA levels were increased by co-administration of sucrose plus acarbose (sucrose + acarbose), but only marginally by that of maltose plus acarbose (maltose + acarbose) (Figure 4E), consistent with our findings in *E. coli* (Figure 2A) and *K. pneumoniae* (Figure 4D). The greater increase in portal SCFA levels induced by sucrose + acarbose than by maltose + acarbose prompted us to measure portal incretin levles. In accordance with our previous finding,^14^ when co-administered with acarbose, sucrose more potently suppressed GIP secretion (Figure 4F) and potentiated GLP-1 secretion (Figure 4G). Taken together, when co-administered with acarbose, sucrose more effectively induces SCFA production by the murine gut microbiome and modulates incretin secretion than maltose.

### Acarbose provides greater protection against diet-induced obesity under high-sucrose feeding than high-maltose feeding

We identified distinct effects of maltose and sucrose, when co-administered with acarbose, on bacterial growth in *E. coli* and *K. pneumoniae* (Figures 2 and 4) and on plasma SCFA and incretin levels in mice (Figures 4E–4G). These findings prompted us to test whether maltose and sucrose differentially influence the gut microbiome and host metabolism during chronic dietary exposure with acarbose. Because microbiota-derived SCFAs exert multiple beneficial effects on the host, including protection against obesity,^11^ we hypothesized that acarbose may influence diet-induced obesity through interactions with dietary disaccharides (maltose or sucrose) and the gut microbiota. As acarbose is an α-GI that suppresses intestinal carbohydrate digestion, we first evaluated its anti-obesity effect in mice fed a disaccharide-free, low-carbohydrate, high-fat (HF) diet (Table S2). Acarbose modestly suppressed body weight gain induced by HF diet over 6 weeks (−4%; Figure S3A). Consistently, acarbose reduced epididymal white adipose tissue (eWAT) and inguinal white adipose tissue (iWAT) weights by 32% and 38%, respectively (Figure S3B), suggesting that α-glucosidase inhibition itself contributes to the suppression of fat accumulation under HF feeding conditions. Consistent with the contribution of α-glucosidase inhibition to the anti-obesity effects of acarbose, supplementation of the HF diet with glucose/fructose (HF + glc/fru) abolished the suppressive effects of acarbose on body weight gain and adipose tissue accumulation (Figures S3C and S3D). We next examined whether dietary maltose further enhances the anti-obesity effects of acarbose. Six weeks of high-fat, high-maltose (HF/HM) diet feeding increased the body weight by 151% compared with the mice fed a normal chow diet (ND), whereas acarbose supplementation reduced this increase to 140% (Figure 5A). This body weight reduction was accompanied by a reduction in the weight of eWAT (−19%) and iWAT (−24%) (Figure 5B). Under *ad libitum* feeding conditions, portal incretin levels tended to decrease for GIP (*p* = 0.3) but significantly increased for GLP-1 (Figure 5C). We subsequently investigated whether sucrose further enhances anti-obesity effects of acarbose using a high-fat, high-sucrose (HF/HS) diet. HF/HS feeding induced body weight gain by 140% compared with that in mice fed ND (Figure 5D), comparable to that observed in the mice fed HF/HM diet (Figure 5A). However, acarbose attenuated HF/HS-induced obesity to a significantly greater extent (−33%) than HF/HM diet-induced obesity (−11%); together with the reduction in diet-induced expansion of eWAT (−77%) and iWAT (−75%) weight (Figure 5E). In addition, the impact of acarbose on decreased GIP levels and increased GLP-1 levels were more pronounced under HF/HS deit feeding than under HF/HM diet (Figure 5F). The effect of long-term supplementation of maltose or sucrose with acarbose on gut microbiota profile was analyzed by fecal 16S rRNA gene sequencing. Compared with ND-fed mice, long-term feeding with HF/HM and HF/HS diets induced similar alterations in gut microbiota composition at the phylum level, characterized by an increased *Firmicutes*-to-*Bacteroidota* ratio (Figure 5G), consistent with previous reports.^29^^,^^30^^,^^31^ Acarbose supplementation of HF/HM and HF/HS (HF/HM + acarbose and HF/HS + acarbose) diets restored these alterations (Figure 5G). Consistent with this compositional shift, acarbose-treated mice exhibited reduced gut microbial α-diversity (Figure 5H). Principal coordinate analysis (PCoA), however, revealed that acarbose-treated groups formed clusters distinct from their respective controls and from each other, indicating compositional differences between HF/HM + acarbose and HF/HS + acarbose at finer taxonomic levels (Figure 5I). To identify bacterial taxa contributing to the compositional differences between HF/HM + acarbose and HF/HS + acarbose diets, we performed linear discriminant analysis effect size (LEfSe).^32^ This analysis identified 7 genera significantly enriched in mice fed HF/HS + acarbose, including *Rikenellaceae* RC9, *Bacteroides*, *Akkermansia*, *Clodtridia* UCG-014, *Parasutterella*, *Muribaculum*, and *Anaerostipes*. In contrast, 8 genera were significantly enriched in mice fed HF/HM + acarbose, including *Parabacteroides*, *Lachnospiraceae* NK4A136, GCA-900066575, *Mucispirillum*, *Lactococcus*, UGC-010, *Butyricicoccaceae*, and A2 (LDA score < 2) (Figure 5J). To further investigate the functional consequences of acarbose-induced microbiome alterations, we performed PICRUSt2-based functional prediction analysis.^33^ We compared the relative abundance of ECs assigned to the KEGG starch and sucrose metabolism pathway (ko00500) between acarbose-treated groups and untreated groups. In HF/HS + acarbose, enzymes involved in starch and sucrose metabolism, including hydrolysis (EC 3.2.1.20) and subsequent phosphorylation (EC 2.7.1.1), were significantly enriched compared with HF/HS (Figure 5K). In contrast, HF/HM + acarbose did not exhibit consistent enrichment of the enzymes (EC 2.4.1.25, EC 2.4.1.8, and EC 3.2.1.122) related to maltose metabolism compared with HF/HM (Figure S3E), suggesting that acarbose selectively enhances microbial metabolic capacity of starch and sucrose, rather than enhancing maltose metabolism under chronic dietary conditions. To identify bacterial taxa contributing to the predicted sucrose metabolic capacity, we further performed PICRUSt2 contribution analysis. *Bacteroides*, which was significantly enriched in HF/HS + acarbose compared with HF/HM + acarbose in LEfSe analysis (Figure 5J), accounted for a substantial proportion of the predicted EC 3.2.1.20 and EC 2.7.1.1 functions, suggesting *Bacteroides* as a candidate contributor to acarbose-associated enhancement of microbial starch and sucrose metabolism (Figure 5L). Based on these findings, we next quantified portal vein SCFA concentrations to determine whether these changes in the gut microbial metabolism can lead to altered SCFA production *in vivo*. Acarbose significantly increased portal vein propionate concentrations in all four dietary conditions (HF/HM, HF/HS, HF, and HF + glc/fru), with the greatest increase observed in HF/HS + acarbose (Figures 5M and S3F). In addition, portal vein butyrate concentrations were significantly increased in HF/HS + acarbose. Notably, the magnitude of the propionate increase in HF + acarbose and HF + glc/fru + acarbose was similar to that observed in HF/HM + acarbose, in which the anti-obesity effect of acarbose was limited, but substantially smaller than that observed in HF/HS + acarbose. Propionate formation by gut microbiota has been reported to occur through three metabolic pathways: the succinate, acrylate, and propanediol pathways.^34^ We therefore examined the metabolic pathways potentially associated with the enrichment of bacterial genera in HF/HS + acarbose. The succinate pathway (P1), involving *Bacteroides*, *Parasutterella*, *Akkermansia*, and *Rikenellaceae* RC9, and the acrylate pathway (P2), involving *Clostridia* UCG-014 and *Anaerostipes*, represented the largest predicted contributions among the genera enriched in HF/HS + acarbose compared with HF/HM + acarbose, suggesting their involvement in the conversion of sucrose-derived substrates to propionate (Figure S4). To identify bacterial taxa at the species level and assess their enrichment in HF/HS + acarbose, we performed BLAST analysis of ASV sequences obtained from 16S rRNA sequencing (Table S1) and subsequently quantified the closest matching bacterial species by species-specific qPCR. This analysis revealed that *Bacteroides caecimuris*, *Bacteroides ovatus*, *Bacteroides sartorii*, *Parasutterella excrementihominis*, and *Akkermansia muciniphila* were significantly enriched in HF/HS + acarbose compared with HF/HM + acarbose, suggesting that these bacterial species are likely to contribute to the conversion of sucrose-derived substrates to propionate. Collectively, these findings indicate that the enhanced anti-obesity effect of acarbose with sucrose involves the gut microbiota-SCFA-incretin axis.

**Figure 5:**
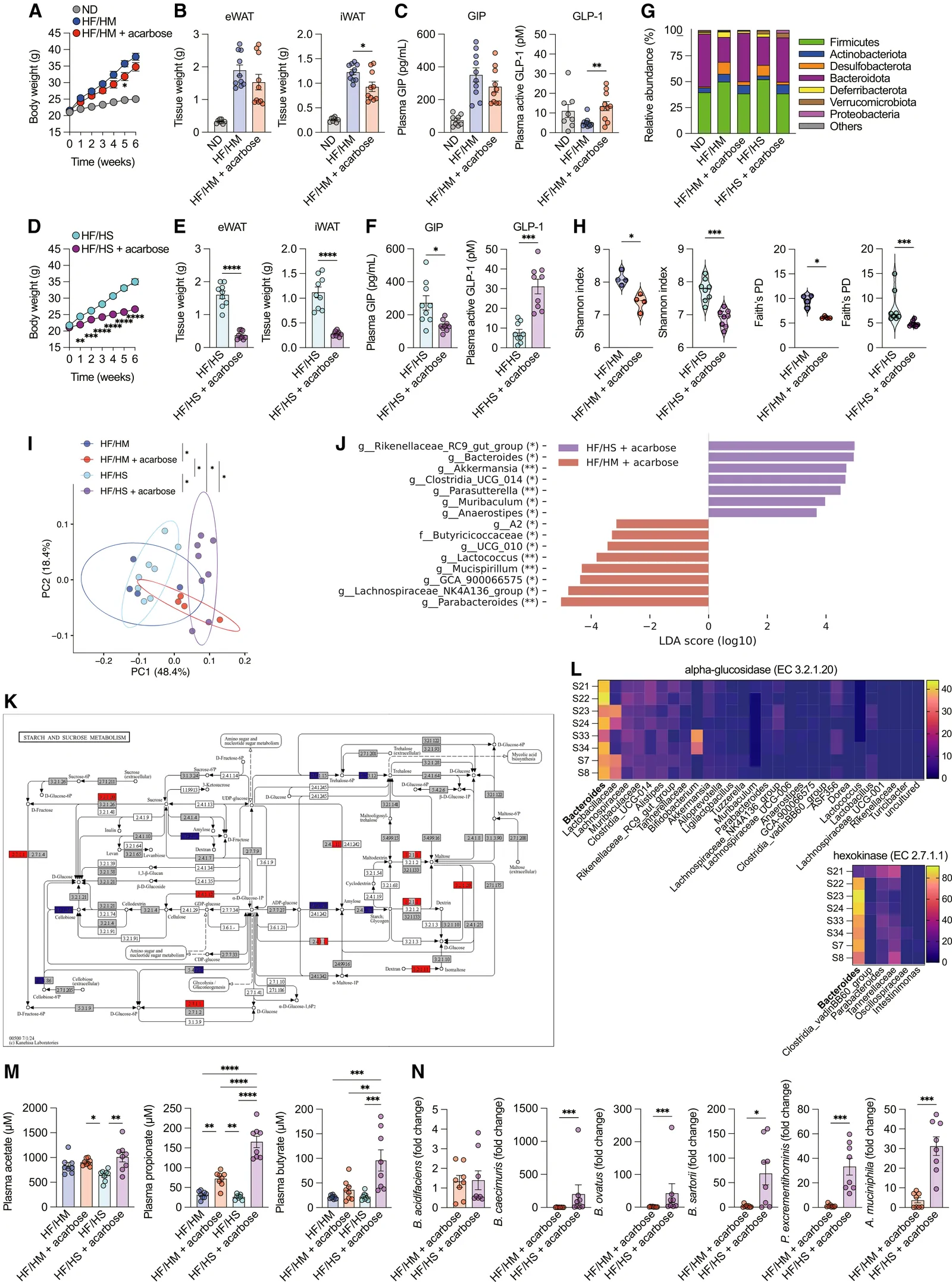
Acarbose provides greater protection against diet-induced obesity under high-sucrose feeding than high-maltose feeding (A–C) Effects of acarbose supplementation on body weight (A), white adipose tissue mass (B), and portal GIP and GLP-1 levels (C) in mice fed an HF/HM diet. Normal diet (ND)–fed mice were included as a reference (*n* = 8–10 biological replicates). (D–F) Effects of acarbose supplementation on body weight (D), white adipose tissue mass (E), and portal GIP and GLP-1 levels (F) in mice fed an HF/HS diet (*n* = 9 biological replicates). Statistical significance for body weight was assessed by mixed-effects model analysis, followed by Šidák multiple comparison test (A and D), whereas adipose tissue mass and GIP and GLP-1 levels were analyzed by unpaired *t* test with Welch’s correction (B, C, E, and F). (G) Relative abundance of major microbial phyla determined by 16S rRNA gene sequencing. Values represent the mean proportion per sample (*n* = 4–8 biological replicates). (H) Gut microbial α-diversity (Shannon index and Faith’s PD) for each group. Differences were assessed using the Mann-Whitney *U* test (*n* = 4–8 biological replicates). (I) Principal coordinate analysis (PCoA) of gut microbiota composition based on Bray-Curtis distances; statistical significance was assessed by pairwise PERMANOVA (*n* = 4–8 biological replicates). (J) Differentially abundant bacterial genera between HF/HM + acarbose and HF/HS + acarbose, identified by LEfSe (Kruskal-Wallis test, ∗*p* < 0.05 and ∗∗*p* < 0.01; LDA score >2) (*n* = 4–8 biological replicates). (K) KEGG pathway map of ko00500 (Starch and sucrose metabolism) showing differentially abundant KEGG orthologs between HF/HS and HF/HS + acarbose based on PICRUSt2-predicted functional profiles (*n* = 8 biological replicates). KEGG orthologs were colored according to log2 fold changes obtained from DESeq2 analysis (adjusted *p* < 0.05), with red indicating enrichment in the HF/HS + acarbose and blue indicating enrichment in the HF/HS. (L) Relative contribution of bacterial genera to EC 3.2.1.20 and EC 2.7.1.1 functions (%) in HF/HS + acarbose samples (*n* = 8 biological replicates), as estimated by PICRUSt2 contribution analysis. (M) Portal SCFA concentrations under *ad libitum* conditions (*n* = 8 biological replicates). Statistical significance was assessed by one-way ANOVA followed by Tukey’s multiple-comparisons test. (N) Species-specific qPCR analysis of fecal bacterial DNA comparing the relative abundance of bacterial species between HF/HM + acarbose and HF/HS + acarbose (*n* = 8 biological replicates). Fold changes were calculated relative to HF/HM + acarbose. ∗*p* < 0.05, ∗∗∗*p* < 0.001, and ∗∗∗∗*p* < 0.0001, as determined by the Mann-Whitney *U* test. Data are presented as mean ± SEM. See also Figures S3 and S4.

## Discussion

α-GIs are widely prescribed for the management of type 2 diabetes to mitigate postprandial hyperglycemia by inhibiting carbohydrate hydrolysis and its absorption.^15^ Beyond this, α-GIs have been reported to modulate incretin secretion,^12^^,^^13^^,^^14^^,^^16^^,^^35^ gut microbial SCFA production,^14^^,^^36^^,^^37^^,^^38^ and alter gut microbial composition and function.^36^^,^^39^^,^^40^^,^^41^^,^^42^ However, how different types of α-GIs and distinct dietary carbohydrates shape host-microbiota responses remains poorly understood. In this study, we show that the α-GI acarbose affects host metabolism not only through inhibition of host carbohydrate-digesting enzymes but also through modulation of the gut microbiome, and that the effect of acarbose varies depending on the type of co-ingested disaccharides.

We demonstrate that acarbose, but not miglitol, blocks SCFA production when the mice are co-administered with maltose, suggesting that acarbose may broadly interfere with gut microbial maltose utilization. Supporting this notion, we found that acarbose, but not miglitol, inhibited bacterial growth on maltose *in vitro*. Specifically, acarbose-induced growth suppression of *E. coli* required LamB, which transports maltose and maltodextrins,^22^ indicating that acarbose inhibits utilization of maltose after being transported into the periplasmic space. Although previous studies have shown that acarbose impairs carbohydrate metabolism in *Bacteroides* species,^43^ and is thought to be transported through LamB^44^ or inhibits cytosolic enzymes,^45^^,^^46^ direct evidence for cytosolic transport of acarbose in living bacteria has been lacking. Our results are consistent with intracellular accumulation of unmetabolized acarbose, leading to inhibition of downstream glycolytic processes in *E. coli*. This effect may contribute to the disruption of MalQ-mediated glucan remodeling and MalP-dependent phosphorolysis, thereby impairing cytosolic maltodextrin metabolism. Although the 2 mM acarbose concentration used in our experiments *in vitro* exceeded the estimated postprandial luminal concentration at the clinical dose (∼0.27 mM, calculated assuming dissolution of a 100 mg dose in the reported postprandial gastric volume of ∼580 mL),^47^ the suppression of growth by acarbose is unlikely to be attributable to nonspecific antibacterial activity. This interpretation is consistent with the lack of growth inhibition in *E. coli* cultured with glucose and *K. pneumoniae* cultured with sucrose. In addition, another α-GI, voglibose, which contains a valiolamine moiety, did not inhibit maltose-dependent growth of E. coli, suggesting that the effect of acarbose might be mediated by the structural properties of acarbose containing a valiolamine moiety. We further identified that the sucrose porin ScrY can transport acarbose. However, acarbose did not inhibit sucrose metabolism in *K. pneumoniae*; instead, it enhanced bacterial growth. Because *Klebsiella grimontii* has been reported to hydrolyze acarbose via an α-glucosidase termed acarbose-preferred glucosidase,^48^ it is possible that *K. pneumoniae* also possesses species-specific mechanisms for acarbose utilization. Consistent with this possibility, acarbose enhanced the growth of *K. pneumoniae* under sucrose conditions without an apparent delay. By contrast, under maltose conditions, it delayed growth but ultimately increased the final yield. These experiments were intended to establish the mechanistic involvement of ScrY in acarbose entry into the cell rather than to model sucrose metabolism in the commensal gut microbiota. Therefore, in this paper, *K. pneumoniae* is used just as a genetic tool for showing ScrY transports acarbose, not a model for healthy gut behavior.

Nonetheless, notably, the carbohydrate-type-dependent effects of acarbose observed in *E. coli* and *K. pneumoniae* were recapitulated in the murine gut microbiota *in vivo*, where acarbose failed to increase SCFA production when co-administered with maltose, but did so with sucrose. Consistent with these findings, the biological impact of acarbose in mammals extends beyond its inhibitory action on host digestive enzymes and includes modulation of incretin secretion, likely secondary to SCFA production by sucrose-metabolizing microbiota. Decreased GIP signaling and increased GLP-1 signaling are considered beneficial for protecting mammals from DIO,^49^^,^^50^ and their secretion might be modulated by SCFA signaling.^12^^,^^13^^,^^14^^,^^16^ In accord with this notion, chronic co-administration of acarbose with sucrose substantially protected the mice from DIO and was associated with elevated portal propionate and butyrate levels, decreased GIP levels, and increased GLP-1 levels. In contrast, these effects of acarbose were largely blunted under HF/HM feeding, suggesting that acarbose-induced differential alterations in gut microbial composition and metabolic function underlie these distinct outcomes.

Obesity is characterized by excessive adipose tissue accumulation driven by a chronic positive energy balance. Substantial evidence indicates that chronic high intake of saturated fats, typically seen in a Westernized diet, contributes to the global rise in obesity prevalence.^51^^,^^52^ High-fat diet feeding is frequently associated with an increased *Firmicutes*-to-*Bacteroidota* (F/B) ratio, although whether this shift represents a cause or consequence of obesity remains debated.^30^^,^^31^ A distinct anti-obesity effect between HF/HM and HF/HS diets, despite a similar effect of acarbose on the F/B ratio, suggested that changes in the F/B ratio are unlikely to contribute to the development of DIO in our study. Alternatively, several bacterial genera, including *Bacteroides*, *Akkermansia*, and *Parasutterella*, were significantly increased by acarbose in the HF/HS but not in the HF/HM, suggesting that these taxa may contribute to the anti-obesity effects. Functional prediction further suggested that acarbose selectively enhanced microbial starch and sucrose metabolism in HF/HS, with *Bacteroides* representing the major predicted contributor to this metabolic capacity, consistent with the sucrose-specific pronounced rise in portal propionate and butyrate observed in HF/HS + acarbose. Brown et al. reported that *Bacteroides ovatus* is less susceptible to acarbose than *Bacteroides thetaiotaomicron* in a starch-based model.^43^ Extending this observation, our species-specific qPCR demonstrated that *B. ovatus* was enriched in HF/HS + acarbose compared with HF/HM + acarbose, suggesting that luminal sucrose retention provides a selective advantage for this species during chronic acarbose treatment. Moreover, Li et al. recently demonstrated that *Parasutterella*-derived propionate mediates the metabolic benefits of acarbose in mice fed a high-fat diet.^53^ Given that *Parasutterella excrementihominis* has been reported to be asaccharolytic^54^ and *Akkermansia muciniphila* preferentially utilizes host-derived mucin rather than dietary carbohydrates,^55^ their enrichment is unlikely to result from direct sucrose utilization. Rather, their enrichment alongside increased portal vein propionate concentrations suggests that acarbose-induced luminal sucrose availability may promote propionate production directly by *Bacteroides* and indirectly by *Parasutterella* and *Akkermansia* through metabolic interactions, potentially involving cross-feeding. Although portal vein butyrate concentrations were markedly elevated in HF/HS + acarbose, the bacterial contributors could not be identified in this study. PICRUSt2 predicted that the unclassified *Clostridia* UCG-014 taxon, which was significantly enriched in HF/HS + acarbose, may contribute to butyrate production from acetyl-CoA generated through downstream sucrose metabolism (Figure S5); however, its species identity and functional capacity remain unresolved. Future studies using gnotobiotic models will be required to determine the causal contributions of individual bacterial taxa and their metabolic interactions to acarbose-mediated anti-obesity effects.

In summary, our findings indicate that inhibition of sucrose digestion by acarbose enables the gut microbiota to metabolize sucrose, thereby increasing microbiota-derived factors, including SCFAs, in mice. The subsequent increase in SCFA signaling is considered to suppress GIP secretion and to increase GLP-1 secretion, collectively contributing to protection against DIO. In addition to dietary fat, excessive intake of refined sugar has been linked to the global rise in obesity,^56^^,^^57^^,^^58^ as supported in our HF/HS and HF/HM models. Notably, the anti-obesity effect of acarbose was more pronounced under a sucrose-rich diet than under a maltose-rich diet, suggesting that sucrose, when co-ingested with acarbose, may act as a prebiotic that enhances microbial fermentation and SCFA production. Once obesity has developed, maintaining self-control over excessive intake of fat and sugar is often difficult.^59^^,^^60^^,^^61^ Therefore, a combination of sucrose and acarbose may provide a promising therapeutic approach for obesity management. Further studies are needed to elucidate the molecular and microbial mechanisms underlying the anti-obesity effects of acarbose under fat- and sucrose-rich dietary conditions.

### Limitations of the study

Several limitations of this study merit consideration. First, although *E. coli* experiments provided proof of concept that acarbose can be taken up in a maltose-dependent manner and suppress bacterial maltose metabolism, *E. coli* does not represent the complexity of the gut microbial community. The relevance of these findings *in vivo* therefore remains to be established. Second, HF/HS + acarbose induced pronounced changes in the gut microbiome, portal SCFA, GIP, and GLP-1 levels, each of which may contribute to the observed anti-obesity effects; however, the causal contribution of each factor could not be fully disentangled. Experiments with germ-free or gnotobiotic mice are required to directly show the involvement of microbiota in these phenotypes. Third, our microbiota analyses support enhanced microbial sucrose metabolism and SCFA production in response to acarbose; however, these findings were based on 16S rRNA sequencing, PICRUSt2-based functional prediction, and species-specific qPCR and therefore do not directly assess microbial transcriptional and enzymatic activity, or metabolic flux. Finally, although acarbose is an established clinical drug, caution is warranted in extrapolating the present findings to humans, as the HF/HS model does not fully recapitulate human Westernized dietary patterns, and the murine gut microbiota differs substantially from that of humans. Further validation in appropriately designed clinical studies is therefore warranted.

## Resource availability

### Lead contact

Requests for further information and resources should be directed to and will be fulfilled by the lead contact, Takashi Miki (tmiki@faculty.chiba-u.jp).

### Materials availability

This study did not generate new unique reagents.

### Data and code availability

• The 16S RNA sequencing and metagenomic profiling datasets generated in this study are available in the NCBI database under BioProject: PRJNA1453709.
• This paper does not report original code.
• Any additional raw data and information to reanalyze the data in this paper are available from the lead contact upon request.

## Acknowledgments

This work was supported by Grant-in-Aid from the Ministry of Education, Science, Sports and Culture of Japan (24K10023 to EL, 20K08583 to RH and 25K02439 to TM), and by Japan Science and Technology Agency (JST) Support for Pioneering Research Initiated by the Next Generation (SPRING), grant no JPMJSP2109 (HY). This work was carried out in part with funding support from Kinugawa Rubber Industrial Co., Ltd. (Chiba, Japan).

## Author contributions

H.Y., E.L., and T.M. designed the study, analyzed the data, and wrote the manuscript. H.Y. and E.L. performed the experiments. R.H. analyzed and interpreted the data. A.T. constructed bacterial mutants and analyzed the data. A.E. conducted metabolome analysis. J.M. and I.K. performed measurements of portal plasma SCFAs. Y.G. conducted bacterial culture experiments. Y.T. and T.T. conducted the computational structural analysis. T.M. is the guarantor of this work and, as such, had full access to all the data in the study and take responsibility for the integrity of the data and the accuracy of the data analysis. All authors reviewed and approved the manuscript.

## Declaration of interests

The authors declare no competing interests.

## STAR★Methods

### Key resources table

**Table:** 

REAGENT or RESOURCE	SOURCE	IDENTIFIER
**Antibodies**		

Anti-RNA polymerase beta antibody	abcam	Cat# EPR18704 RRID: AB_3076167
LAMB Polyclonal Antibody	Thermo Fisher Scientific (Invitrogen)	Cat# PA5-144472 RRID: AB_3091635
Anti-rabbit IgG, HRP-linked Antibody	Cell Signaling Technology	Cat# 7074 RRID: AB_2099233

**Bacterial and virus strains**		

*E. coli* DH5α	Our collection	N/A
*E. coli* W3110	Our collection	N/A
*K. pneumoniae* Kpc01	Yamanaka et al.^28^	N/A
*E. coli* W3110 *ΔlamB*	This paper	N/A
*E. coli* W3110 *ΔlamB/pscrY*	This paper	N/A
*E. coli* W3110 *ΔlamB/plamBEc*	This paper	N/A
*E. coli* W3110 *ΔlamB/plamBKp*	This paper	N/A

**Biological samples**		

Mouse plasma	This paper	N/A
Mouse feces	This paper	N/A
Mouse adipose tissue	This paper	N/A

**Chemicals, peptides, and recombinant proteins**		

Acarbose	Tokyo Chemical Industry	Cat# A2485
Miglitol	Tokyo Chemical Industry	Cat# M2302
Voglibose	Tokyo Chemical Industry	Cat# V0119
Maltotriose	Cayman Chemical	Cat# 20306
Maltotetraose	Nagase Viita Co., Ltd.	Cat# MA141
Maltohexaose	Apollo Scientific	Cat# BIM1013
Diprotin A	Peptide Institute Inc.	Cat# 4132
Fast SYBR Green Master Mix	Applied Biosystems	Cat# 4385612

**Critical commercial assays**		

Active GLP-1 assay kit	Millipore	Cat# EGLP-35K
Rat/Mouse Total GIP (Total) ELISA kit	Millipore	Cat# EZRMGIP-55K
Enzytec^TM^ Liquid Acetic acid assay kit	R-Biopharm	Cat# E8226

**Deposited data**		

16S rRNA sequencing (raw sequence data)	This paper	BioProject: PRJNA1453709

**Experimental models: Organisms/strains**		

Mouse: C57BL/6J	CLEA Japan Inc.	N/A

**Oligonucleotides**		

Oligonucleotides sequences are listed in Tables S3 and S4	This paper	N/A

**Software and algorithms**		

QIIME 2	QIIME 2	Version 2024.10
PICRUSt2	Douglas et al.^33^	Version 2.5.3
GraphPad Prism 10	GraphPad Software, LLC.	Version 10.4.1
R	R Foundation for Statistical Computing	Version 4.4.0
R studio	Posit	Version 2026.05.0 + 218
MS-DIAL	Tsugawa et al.^62^	Version 4.9.221218

**Other**		

Mouse: Normal chow diet	CLEA Japan Inc.	CE-2
Mouse: High-fat, high-sucrose diet (45% kcal from fat)	Research Diet Inc.	D12451
Mouse: High-fat, high-maltose diet (45% kcal from fat)	Research Diet Inc.	N/A
Mouse: High-fat diet (54% kcal from fat)	Research Diet Inc.	N/A
Mouse: High-fat, fructose/glucose diet	This paper	N/A
Fusion imaging station	Vilber Lourmat	FUSION-FX7

### Experimental model and study participant details

#### Experimental model

##### Animals

Wild-type male C57BL/6J mice were purchased from CLEA Japan, Inc. (Tokyo, Japan). The mice were housed in a climate-controlled room with a temperature of 23 ± 3 °C, humidity of 55 ± 15%, and a 12 h light/12 h dark cycle and provided *ad libitum* access to food and water. Mice were assigned to one of the following diets: a normal chow diet (ND) (CE-2; 12.1% kcal from fat; CLEA Japan); a high-fat, high-sucrose diet (HF/HS) (45% kcal from fat and 17% kcal from sucrose; D12451; Research Diets, Inc., New Brunswick, NJ, USA); a high-fat, high-maltose diet (HF/HM) (45% kcal from fat and 17% kcal from maltose; D22122002, modified D12451 formula); a high-fat diet (HF) (54% kcal from fat; D12451px13, modified D12451 formula); or a high-fat diet supplemented with an equimolar mixture of glucose and fructose (HF + glc/fru) (45% kcal from fat and 8.5% kcal each from glucose and fructose). The α-GI acarbose (1,000 ppm) (Tokyo Chemical Industry Co., Ltd., Tokyo, Japan) was incorporated into the HF/HS (HF/HS + acarbose), HF/HM (HF/HM + acarbose), HF (HF + acarbose), or HF + glc/fru (HF + glc/fru + acarbose) diets. The nutritional composition of all experimental diets except ND is provided in Table S2. Diet interventions were initiated at 6 weeks of age and were maintained for 6 weeks. Sample sizes for each experiment are indicated in the corresponding figure legends. All animal experiments were approved by the Animal Care and Use Committee of Chiba University, Japan (Approval No. 7–139 and 8–105), and were conducted in accordance with institutional guidelines.

##### Bacterial strains

*Klebsiella pneumoniae* Kpc01 is a bloodstream clinical isolate from our collection.^28^ *K. pneumoniae* Kpc01 and *Escherichia coli* W3110 were used for mutant construction and competition assay, whereas *E. coli* DH5α was used for competition assay and routine plasmid cloning. Unless otherwise stated, strains were grown overnight in LB (Invitrogen, Life Technologies, Carlsbad, CA, USA) broth at 37 °C with vigorous shaking. No antibiotics were added during routine cultivation. When maintaining plasmids, ampicillin was used at 50 μg/mL. Bacterial strains were preserved as 15% glycerol stocks at −80°C. All strains used in this study are listed in Table 1.

### Method details

#### Portal incretin measurements in the fed state

After 6 weeks of dietary intervention, mice were anesthetized with isoflurane and portal blood (∼600 μL) was collected at 10:00 a.m., corresponding to the fed state (4 h after light onset). Blood samples were immediately mixed with EDTA (final concentration, 0.15%) and diprotin A (final concentration, 3 mM) (Peptide Institute Inc., Osaka, Japan) to prevent peptide degradation. Plasma was separated by centrifugation (2,000×g, 15 min, 4 °C) and stored at −80°C until analysis. Plasma incretin concentrations were measured using a Glucagon-Like Peptide-1 (Active) ELISA (Millipore, Billerica, MA, USA) and a Rat/Mouse Total GIP ELISA (Millipore, Billerica, MA, USA), according to the manufacturer’s instructions.

#### Acute incretin secretion in response to disaccharides challenge

After a 16 h fast, disaccharides (maltose or sucrose; 2 g/kg) were orally administered with or without the acarbose (40 mg/kg). Portal blood was collected 30 min after administration, and plasma GIP and GLP-1 levels were measured.

#### Portal short-chain fatty acid (SCFA) measurements *in vivo*

Portal SCFA concentrations were measured 30 min after oral administration of maltose or sucrose (2 g/kg) with or without α-GIs (acarbose, 40 mg/kg, or miglitol, 10 mg/kg; Tokyo Chemical Industry Co., Ltd., Tokyo, Japan). These doses were selected based on a preliminary oral maltose tolerance test (Figure S1). For the maltose challenge experiment, the primary comparison was between the maltose + acarbose and maltose + miglitol groups (*n* = 8 per group), whereas the maltose-alone group (*n* = 4) was included to assess the inhibition of glycemic response after maltose administration. For the sucrose and maltose comparison, the primary comparison was between the sucrose + acarbose and maltose + acarbose groups (*n* = 12 per group). In addition, portal SCFA concentrations were measured after 6 weeks of dietary intervention using samples collected at 10:00 a.m. (fed state; 4 h after light onset; *n* = 8 per group). Data for the vehicle and maltose + miglitol groups were the same data as reported in our previous study (Ma et al., 2022),^17^ and are presented here for comparison. Although these data have been published previously,^17^ they originate from the same experiment as the current data and are reproduced here solely for the purpose of comparison in Figure 1B. SCFA levels were quantified by gas chromatography-mass spectrometry (GC-MS; GCMS-QP2010 Ultra, Shimadzu, Kyoto, Japan) as previously described.^63^ Briefly, chromatographic separation was performed using a VF-WAXms capillary column (30 m × 0.25 mm i.d., 1 μm film thickness; Agilent Technologies) with helium as the carrier gas (0.92 mL/min). Mass spectrometry was performed in scan mode (m/z 40–130) and selected ion monitoring mode for SCFA quantification using external standard calibration.

#### Construction of *E. coli* W3110 ΔlamB strain

The *E. coli* W3110 *ΔlamB* mutant was generated using the λ-Red recombination system.^64^ *E. coli* W3110 was transformed with pKD46 and maintained in LB supplemented with ampicillin (50 μg/mL) at 30 °C. To induce λ-Red recombination, cultures were grown in the presence of 0.2% (w/v) L-arabinose to an optical density at 600 nm (OD600) of approximately 0.5, and electrocompetent cells were prepared. A linear DNA fragment carrying a chloramphenicol resistance cassette was PCR-amplified from pKD3 using the primer pair lamB-P1-F (5′-acacaaagcctgtcacaggtgatgtgaaaaaagaaaagcaatgactcagggtgtaggctggagctgcttc-3′)/lamB-P1-F (5′-ggcgttggttgccgaatgcggcgtaaacgccttatccggcccaggttttgcatatgaatatcctccttag-3′). The PCR product was introduced into the induced cells by electroporation, and recombinants were selected on LB agar containing chloramphenicol (20 μg/mL) at 37 °C. Correct integration at the target locus was confirmed by colony PCR using primers lamB-check-F (5′-ggtgccacattcgctatcgg-3′) and lamB-check-R (5′-acatttgacagccgttgtag-3′). For marker excision, pCP20 was introduced into recombinant strains to remove the chloramphenicol cassette and cassette loss was verified by chloramphenicol susceptibility and PCR.

#### Plasmid construction

The coding sequences of srcY and lamB from *E. coli* W3110 and lamB from *K. pneumoniae* Kpc01 were amplified by PCR using the primer pairs KpnI-srcY-F (5′-ccgggtaccagcacactcactgatttacc-3′)/EcoRI-srcY-R (5′-agtgaattccacgtcagaaccaagtctcc-3′), KpnI-EclamB-F (5′-ccgggtaccagcctgtcacaggtgatgtg-3′)/EcoRI-EclamB-R (5′-agtgaattcgctattaccaccagatttcc-3′), and KpnI-Kp_lamB-F (5′-ccgggtacccgatataccggcgaagcgtg-3′) and EcoRI-Kp_lamB-R (5′-agtgaattcggtattaccaccacacttcc-3′), respectively. PCR products were ligated into vector pMW119 to generate pMW119-srcY (psrcY), pMW119-lamBEc (plamBEc), and pMW119-lamBKp (plamBKp). The resulting plasmids were introduced into the *E. coli ΔlamB* strain.

#### Culturing of *E. coli* in various types of sugar

Overnight cultures of *E. coli* were resuspended in M9 medium to an OD600 of 0.1. The M9 medium was supplemented with 0.2% (w/v) thiamine, 2 mM MgSO_4_, and 0.1 mM CaCl_2_, and, where indicated, 0.5% (w/v) maltose, maltotriose, maltotetraose, maltohexaose, glucose, or sucrose was added as the carbon source. Experiments were performed in the presence or absence of α-GIs (2 mM miglitol, 2 mM acarbose, or 2 mM voglibose). Cultures were incubated at 37 °C with shaking, and OD600 values were measured at the time points indicated in the figures.

#### Measurement of acetate in culture medium

To assess acetate production by *E. coli* and *K. pneumoniae*, culture samples were collected and centrifuged at 2,000×g for 10 min at 4 °C. The resulting supernatants were used to determine acetate concentrations using an acetic acid test kit (R-Biopharm AG, Darmstadt, Germany), according to the manufacturer’s instructions.

#### Quantitative reverse transcription polymerase chain reaction (RT-qPCR) analyses

*E. coli* W3110 was cultured in M9/maltose medium for 6 h, washed, and subsequently incubated in carbohydrate-free M9 medium containing acarbose alone (2 mM or 25 mM) at 37 °C for 15 min. Total RNA was extracted from each culture using ISOGEN (FUJIFILM Wako Pure Chemical Corporation, Osaka, Japan). Complementary DNA (cDNA) was synthesized and subjected to quantitative PCR (qPCR) using Fast SYBR Green Master Mix (Applied Biosystems, Waltham, MA, USA), according to the manufacturer’s instruction. Expression of genes in maltose operon was analyzed by RT–qPCR. Primer sequences are listed in Table S3.

#### Mass spectrometry analysis

After overnight culture in LB broth, *E. coli* W3110 in M9/maltose was precultured for 6 h. Cells were then washed once and transferred to one of the following three conditions: M9 medium without a carbon source (M9/-), M9/maltose, or M9/maltose supplemented with acarbose. Cells with OD600 0.1 were then incubated for 1 h, harvested, and flash-frozen in liquid nitrogen and stored at −80°C until extraction. Three independent biological replicates were prepared for each condition (*n* = 3). Metabolites were extracted using a modified protocol based on that described by Carvalho et al.^65^ Frozen pellets were resuspended in ice-cold (−20°C) 80% (v/v) methanol at a concentration corresponding to 200 mg wet cell weight per mL. Stainless-steel beads (0.6 mm diameter) were added, and cells were disrupted by five cycles of vigorous vortexing for 30 s followed by incubation on ice for 30 s. Debris was removed by centrifugation (4,000×g, 10 min, 4 °C), and supernatants were subjected to ultra-performance liquid chromatography–quantitative time-of-flight mass spectrometry (UPLC–QTOF-MS) analysis as previously described (Eguchi et al.^66^; Takeda et al.^67^). Analyses were performed using an ExionLC AD UPLC system coupled to an X500R LC-QToFMS system (SCIEX) with Sequential Windowed Acquisition of All Theoretical Fragment Ion Mass Spectra (SWATH) method. A duty cycle included a single MS scan (200 ms) followed by a series of product ion scans (40 ms each) across 30 isolation windows (m/z 50–800). MS/MS spectra were acquired at 35 eV (±15 eV). Chromatographic separation was conducted using an Agilent InfinityLab Poroshell 120 HILIC-Z column (2.7 μm, 50 × 2.1 mm) under previously reported gradient conditions.^17^^,^^61^ Ionization was performed in both positive and negative modes. Data processing was performed using MS-DIAL (ver. 4.9.221218)^62^ and R (ver. 4.4.0). For relative quantification, the peak area of each identified metabolite was used for comparison between groups. Spectra were matched against public libraries, including MS-DIAL MSP, MassBank of North America, and NIST20. Metabolite annotations were assigned to Level 2 according to Schymanski et al.,^68^ based on the highest-scoring matches in spectral libraries with a cutoff score of 80.^66^ Among the detected glucans, maltopentaose was identified at Level 2 by direct spectral matching, whereas other oligomers were assigned to Level 3 based on their MS/MS features and characteristic mass differences corresponding to hexose units (162.05 Da), without resolving specific glycosidic linkages. Acarbose identification was confirmed using an authentic standard.

#### Western blotting

*E. coli* W3110, *ΔlamB*, and *ΔlamB/lamB*_*Ec*_ strains were cultured in M9 minimal medium supplemented with 0.5% (w/v) maltose or glucose for 6 h to reach mid-log phase. Cells were harvested by centrifugation and lysed in Laemmli sample buffer, followed by sonication and boiling at 100 °C for 5 min. Protein lysates (5 μL) were separated by SDS–polyacrylamide gel electrophoresis and transferred onto polyvinylidene difluoride (PVDF) membranes. Membranes were incubated with primary antibodies against RpoB (EPR18704, Abcam; 1:2,000) and LamB (PA5-144472, Thermo Fisher Scientific; 1:2,000), followed by HRP-conjugated anti-rabbit secondary antibodies (#7047, Cell Signaling Technology; 1:10,000). Immunoreactive signals were detected and quantified using a FUSION-FX7 imaging system (Vilber Lourmat, Collégien, France).

#### Docking simulations

The crystal structure of MalQ complex with acarviosine-glucose-acarbose (AGA) (PDB 4S3R) and MalP complex with alpha-acarbose (PDB 2ECP) were used. Molecular docking between MalQ or MalP and maltooligosaccharids or acarbose were performed using AutoDock Vina (AutoDockTools v1.5.6). Docking scores are reported as binding energies (kcal/mol), with lower values indicating more stable interactions; the lowest-energy pose was selected for further analysis. Predicted hydrogen bonds or distance between ligands and amino acid residues were identified based on geometric criteria, and the corresponding distances (Å) were measured. Molecular graphics and visualization were generated in using PyMOL (Schrödinger, LLC).

#### Gut microbial composition analyses by 16S rRNA gene sequencing

Feces were collected immediately after defecation at the end of the long-term dietary intervention, immediately frozen, and stored at −80°C until analysis. Total bacterial DNA was extracted using the FastDNA SPIN Kit for Feces (MP Biomedicals, CA, USA) according to the manufacturer’s instructions. The v4 hypervariable region of the bacterial 16S rRNA gene was amplified by PCR using primers 515F (5′-TCGTCGGCAGCGTCAGATGTGTATAAGAGACAGGTGYCAGCMGCCGCGGTAA-3′) and 806R (5′-GTCTCGTGGGCTCGGAGATGTGTATAAGAGACAGGGACTACHVGGGTWTCTAAT-3′). Amplicons were purified using AMPure XP beads (Beckman Coulter, CA, USA) and subjected to paired-end sequencing on an Illumina MiSeq platform (Illumina, CA, USA) using the MiSeq Reagent Kit v3. Raw sequencing data were processed using QIIME 2 (version 2024.10). Sequences were quality-filtered, denoised, and merged to generate amplicon sequence variants (ASVs). Taxonomic assignment was performed using the SILVA 138 database (silva-138–99-nb-classifier). Alpha-diversity metrics and beta-diversity analysis based on Bray–Curtis distances were calculated within QIIME 2, and principal coordinate analysis (PCoA) was used for visualization. Differentially enriched taxa were identified using linear discriminant analysis effect size (LEfSe) as described in the main text.

#### PICRUSt2-based functional prediction analysis

Functional profiles of the gut microbiota were predicted from 16S rRNA gene sequencing data using PICRUSt2 (v2.5.3).^33^ Predicted KEGG ortholog (KO) abundances were compared between HF/HS and HF/HS + acarbose mice, and between HF/HM and HF/HM + acarbose mice using DESeq2. Differentially abundant KOs within the KEGG starch and sucrose metabolism (ko00500) pathway were identified and visualized using KEGG Mapper. Permission to reproduce the KEGG pathway image was obtained from Kanehisa Laboratories. The information presented in the KEGG map is based on the KEGG database.^69^ To identify bacterial taxa contributing to the predicted metabolic functions in HF/HS + acarbose samples, PICRUSt2 contribution analysis was performed using the *pred_metagenome_contrib.tsv* output. For EC 3.2.1.20 and EC 2.7.1.1 involved in sucrose metabolism, normalized taxon function contributions were aggregated at the genus level, and the relative contribution of each genus was calculated. For ECs associated with the known propionate-producing pathways^34^ and butyrate-producing pathways^70^ contribution values were aggregated at the genus level, and the relative contribution of each taxon was calculated.

#### Species-specific quantitative PCR analysis of gut microbiota

Species-specific quantitative PCR (qPCR) was performed to validate bacterial taxa identified by 16S rRNA gene sequencing analysis. Representative amplicon sequence variants (ASVs) assigned to genus *Bacteroides*, *Parasutterella*, and *Akkermansia* were subjected to BLAST analysis to identify the closest matching bacterial species. The corresponding ASV sequences and sequence identities are summarized in Table S1. qPCR was performed using bacterial DNA extracted from fecal samples, and the relative abundance of each bacterial species was calculated using the ΔΔCt method normalized to universal bacterial 16S rRNA gene amplification. Species-specific primer sequences were listed in Table S4. Relative changes in bacterial abundance were indicated as fold changes in HF/HS + acarbose mice relative to HF/HM + acarbose mice.

### Quantification and statistical analysis

All quantitative data are presented as mean ± SEM, unless otherwise indicated. Statistical analyses were performed using GraphPad Prism software (version 8.0). Comparisons between two groups were conducted using an unpaired two-tailed Student’s *t* test. Welch’s correction was applied when variances were unequal. Species-specific bacterial qPCR data were analyzed using the Mann–Whitney *U* test because the relative abundance data did not satisfy the assumptions of parametric testing. Comparisons involving more than two groups were performed using one-way analysis of variance (ANOVA) followed by Tukey’s multiple-comparisons test or, where appropriate, the Kruskal–Wallis test followed by Dunn’s multiple-comparisons test. Statistical details, including sample sizes (*n*), the definition of *n*, statistical tests, and measures of center, dispersion, and precision, are provided in the corresponding figure legends. A *p* value <0.05 was considered statistically significant.

## Supplemental information

Document S1. Figures S1–S5 and Tables S2–S4, Data S1, and supplemental references

Table S1. BLAST-based identification of bacterial species from ASV sequences, related to Figure 5
