# Supplementary material for: Acarbose differentially affects gut microbial metabolism of maltose and sucrose, distinctly modulating host metabolism
Source: iScience. 2026 Oct 13;29(11):117889. doi: 10.1016/j.isci.2026.117889 (PMC13683718; doi:10.1016/j.isci.2026.117889)
Supplement: Supplementary file 1 [file mmc1.pdf]

iScience, Volume *29*

Supplemental information

Acarbose differentially affects gut microbial metabolism of maltose and sucrose, distinctly modulating host metabolism

Hayato Yoshikawa, Eunyoung Lee, Ryo Hatano, Yuki Taki, Akiko Takaya, Akifumi Eguchi, Yoshiyuki Goto, Junki Miyamoto, Ikuo Kimura, Tomoaki Tanaka, and Takashi Miki

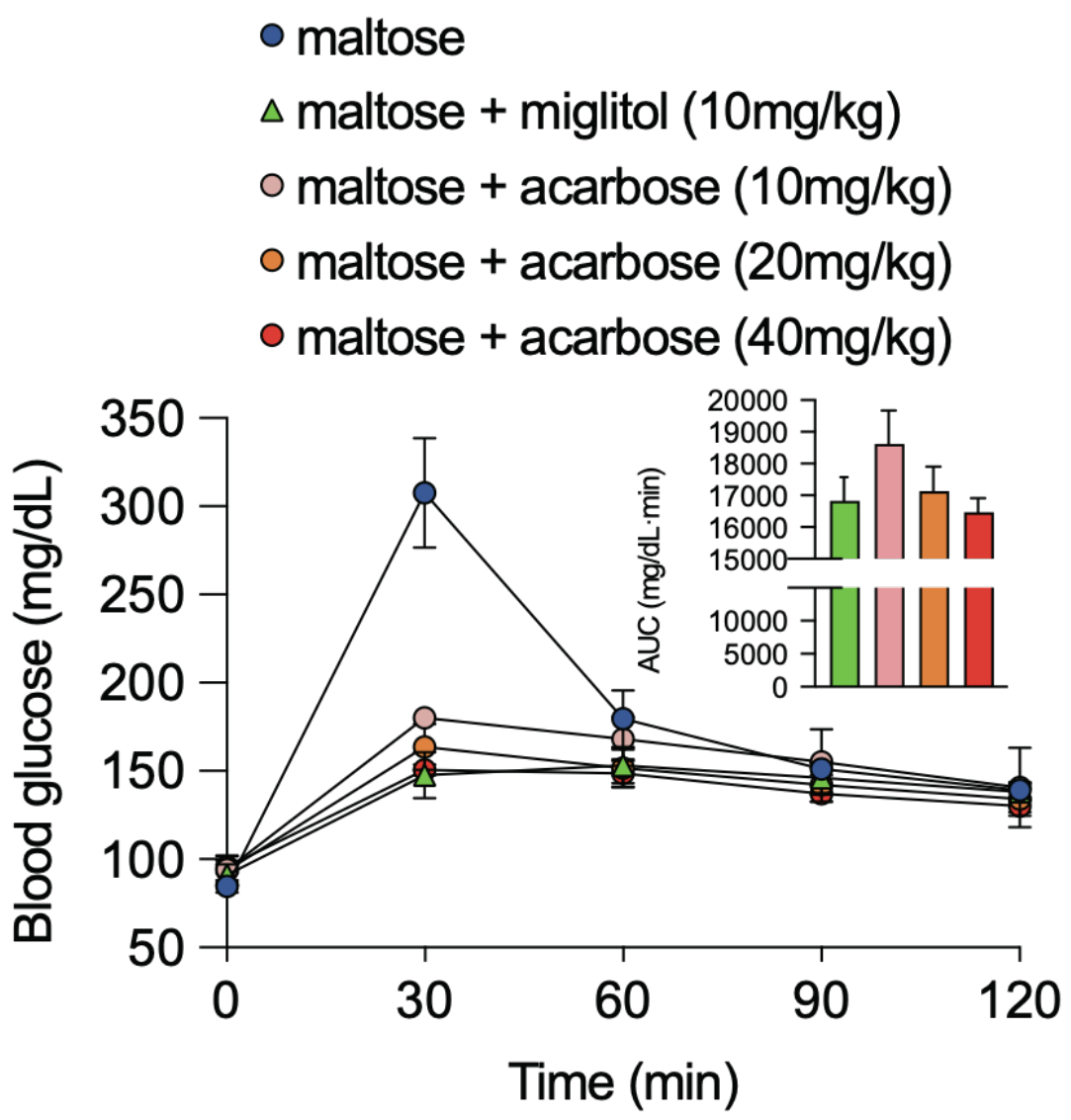

Figure S1. Effects of miglitol and acarbose on blood glucose levels during an oral maltose tolerance test, related to Figure 1.

Blood glucose levels at 0, 30, 60, 90, and 120 min following oral administration of maltose (2 g/kg) with miglitol (10 mg/kg) or acarbose (10, 20, or 40 mg/kg). The area under the blood glucose concentration–time curve (AUC) is also shown. Data are presented as mean ± SEM (*n* = 3–4).

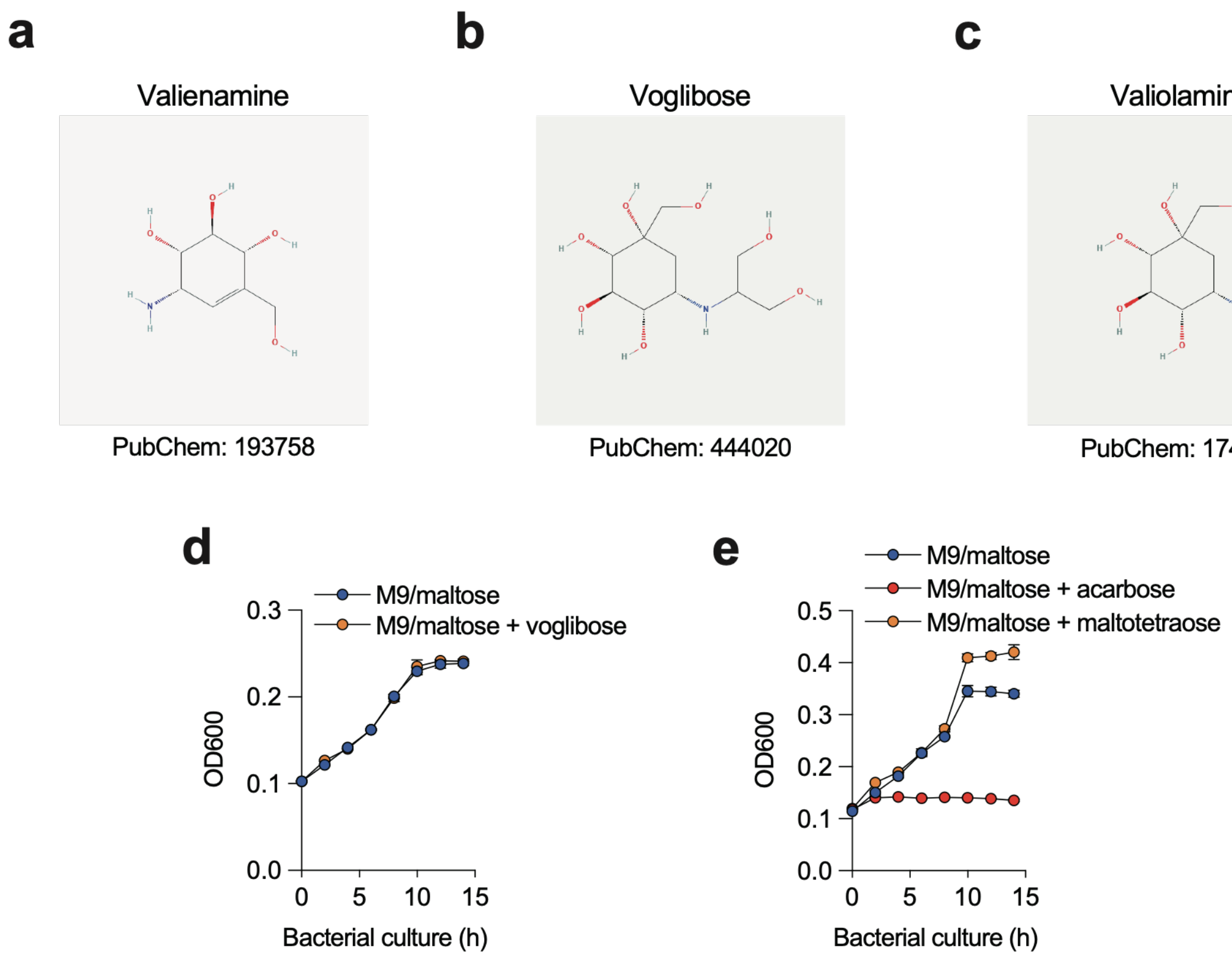

Figure S2. Structures of acarbose-related compounds and effects on E. coli growth in M9/maltose medium, related to Figure 2.

a–c Chemical structures of valienamine (**a**), voglibose (**b**), and valiolamine (**c**). Valienamine and valiolamine constitute the pharmacophore moieties of acarbose and voglibose, respectively. These structures are shown to illustrate the structural basis of these α-glucosidase inhibitors. **d** Effect of 2 mM voglibose on *E. coli* growth in the presence of maltose (*n* = 3). **e** Effect of 2 mM maltotetraose on *E. coli* growth in the presence of maltose (*n* = 4). Data are presented as mean ± SEM.

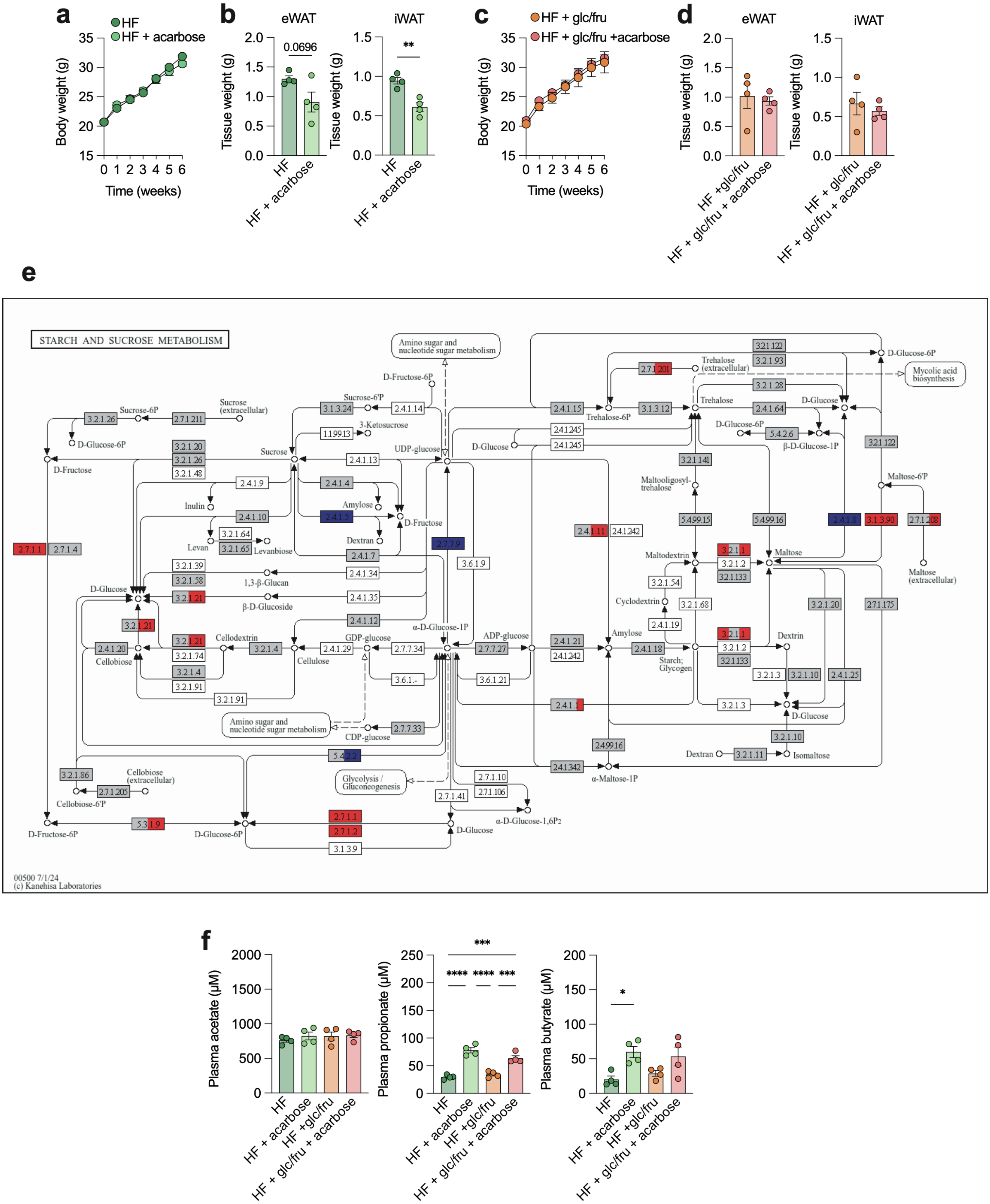
a
HF
HF + acarbose
Body weight (g)
Time (weeks)
b
eWAT
iWAT
0.0696
**
Tissue weight (g)
HF
HF + acarbose
c
HF + glc/fru
HF + glc/fru +acarbose
Body weight (g)
Time (weeks)
d
eWAT
iWAT
Tissue weight (g)
HF +glc/fru
HF + glc/fru + acarbose
e
STARCH AND SUCROSE METABOLISM
Amino sugar and nucleotide sugar metabolism
Mycolic acid biosynthesis
Glycolysis / Gluconeogenesis
Sucrose
Sucrose-6P
Sucrose (extracellular)
D-Fructose
D-Fructose-6P
D-Glucose
D-Glucose-6P
UDP-glucose
ADP-glucose
GDP-glucose
CDP-glucose
α-D-Glucose-1P
α-D-Glucose-1,6P2
Trehalose
Trehalose-6P
Trehalose (extracellular)
Maltose
Maltose-6'P
Maltose (extracellular)
Maltodextrin
Maltooligosyl-trehalose
Cyclodextrin
Amylose
Starch; Glycogen
Dextrin
Dextran
Isomaltose
α-Maltose-1P
β-D-Glucose-1P
Cellobiose
Cellobiose-6'P
Cellobiose (extracellular)
Cellodextrin
Cellulose
Levan
Levanbiose
Inulin
3-Ketosucrose
1,3-β-Glucan
β-D-Glucoside
00500 7/1/24
(c) Kanehisa Laboratories
f
Plasma acetate (μM)
Plasma propionate (μM)
Plasma butyrate (μM)
***
****
***
*
HF
HF + acarbose
HF +glc/fru
HF + glc/fru + acarbose

Figure S3. Effects of acarbose on diet-induced models, gut microbial carbohydrate metabolism, and circulating short-chain fatty acids, related to Figure 5.

a,b Effects of acarbose on body weight (**a**) and white adipose tissue (WAT) weight (**b**) in mice fed a high-fat (HF) diet (n = 4). **c,d** Effects of acarbose on body weight (**c**) and WAT weight (**d**) in mice fed a high-fat diet supplemented with glucose and fructose (HF + glc/fru) (n = 4). Data are presented as mean ± SEM. p values from an unpaired t-test: **$p < 0.01$. **e** Effects of acarbose on the predicted gut microbial starch and sucrose metabolism pathway (KEGG orthology ko00500) in mice fed an HF/HM diet, as predicted by PICRUSt2 (n = 4). KEGG orthologs were colored according to log2 fold changes obtained from DESeq2 analysis (adjusted $p < 0.05$), with red indicating enrichment in the HF/HM + acarbose and blue indicating enrichment in the HF/HM. **f** Portal short-chain fatty acid levels in mice fed HF or HF + glc/fru diets with or without acarbose (n = 4). Data are presented as mean ± SEM. *$p < 0.05$, ***$p < 0.001$, ****$p < 0.0001$, as determined by one-way ANOVA followed by Tukey's multiple-comparisons test.

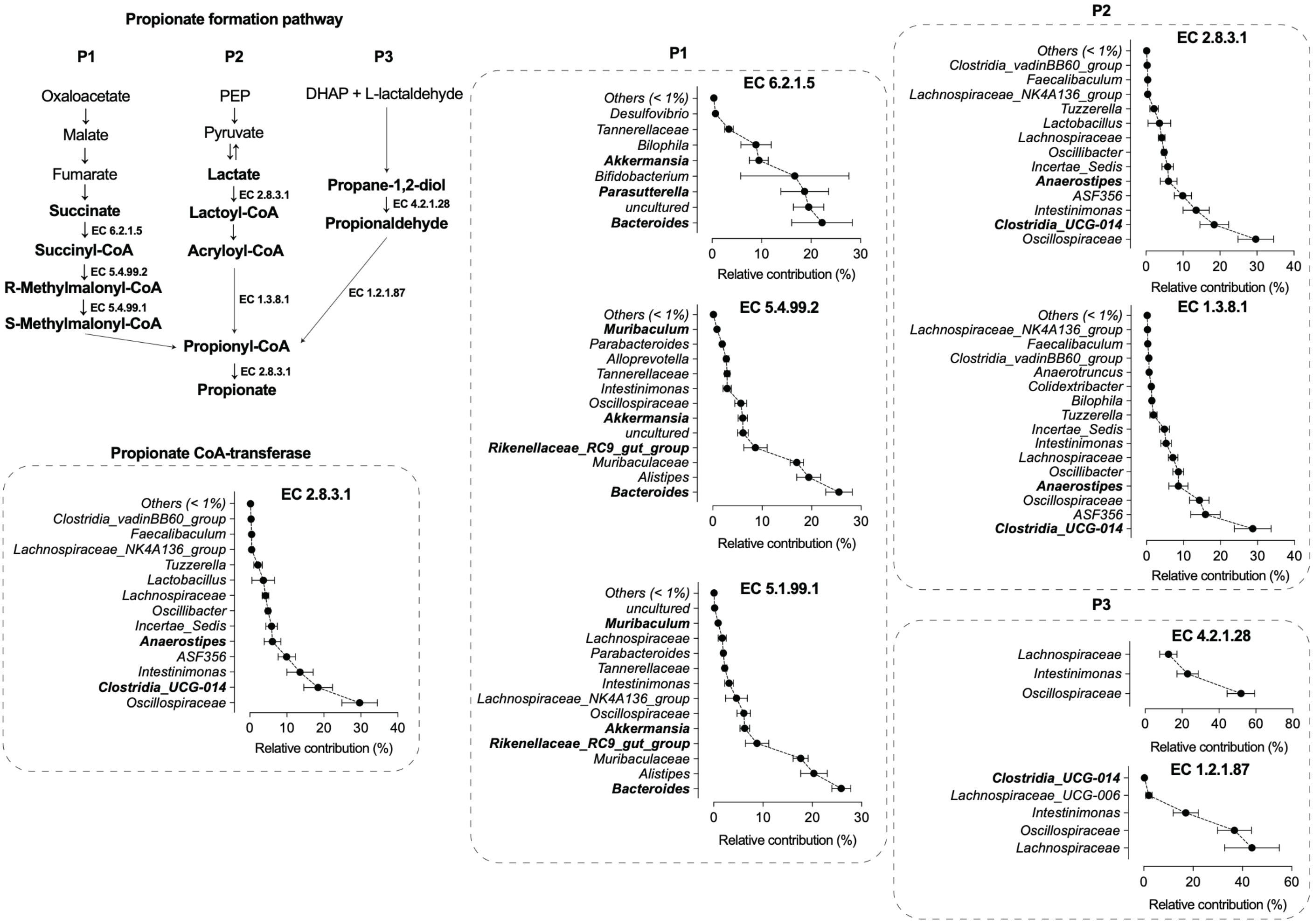

Figure S4. Predicted bacterial contribution to gut microbial propionate production pathway, related to Figure 5.

Propionate production pathways were categorized into the succinate (P1), acrylate (P2), and propanediol (P3) pathways based on Reichardt *et al.*[33] EC numbers corresponding to each pathway were identified from the KEGG propanoate metabolism pathway (ko00640). Using 16S rRNA gene sequencing data from HF/HS + acarbose, PICRUSt2 contribution analysis was performed to estimate the contribution of individual bacterial genera to each EC. The relative contribution of each bacterial genus is expressed as a percentage of the total predicted contribution to the respective EC. Data are presented as mean ± SEM (*n* = 8).

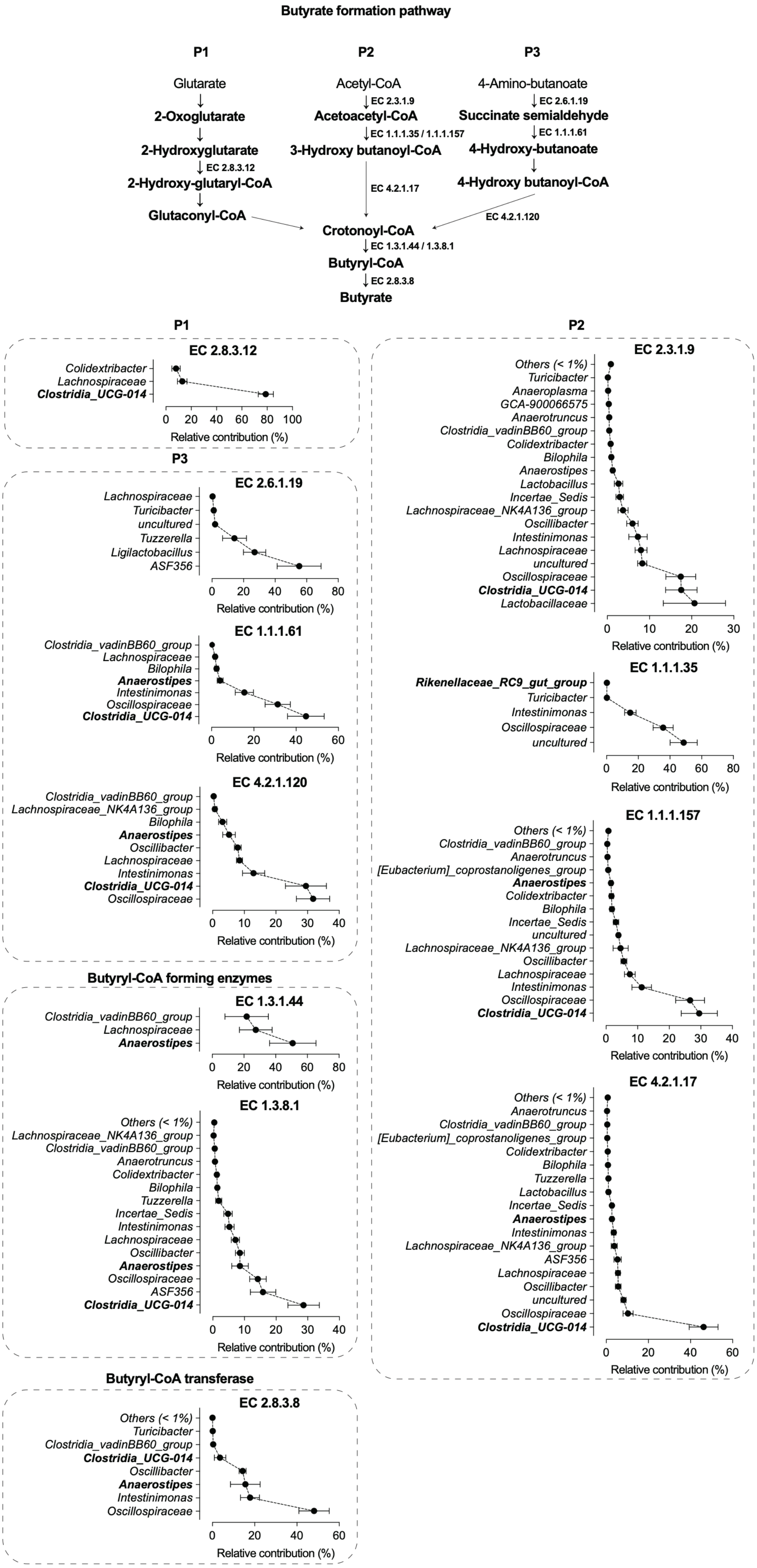

Butyrate formation pathway
P1
P2
P3
Glutarate
2-Oxoglutarate
2-Hydroxyglutarate
EC 2.8.3.12
2-Hydroxy-glutaryl-CoA
Glutaconyl-CoA
Acetyl-CoA
EC 2.3.1.9
Acetoacetyl-CoA
EC 1.1.1.35 / 1.1.1.157
3-Hydroxy butanoyl-CoA
EC 4.2.1.17
4-Amino-butanoate
EC 2.6.1.19
Succinate semialdehyde
EC 1.1.1.61
4-Hydroxy-butanoate
4-Hydroxy butanoyl-CoA
EC 4.2.1.120
Crotonoyl-CoA
EC 1.3.1.44 / 1.3.8.1
Butyryl-CoA
EC 2.8.3.8
Butyrate
P1
EC 2.8.3.12
Colidextribacter
Lachnospiraceae
Clostridia_UCG-014
0 20 40 60 80 100
Relative contribution (%)
P3
EC 2.6.1.19
Lachnospiraceae
Turicibacter
uncultured
Tuzzerella
Ligilactobacillus
ASF356
0 20 40 60 80
Relative contribution (%)
EC 1.1.1.61
Clostridia_vadinBB60_group
Lachnospiraceae
Bilophila
Anaerostipes
Intestinimonas
Oscillospiraceae
Clostridia_UCG-014
0 20 40 60
Relative contribution (%)
EC 4.2.1.120
Clostridia_vadinBB60_group
Lachnospiraceae_NK4A136_group
Bilophila
Anaerostipes
Oscillibacter
Lachnospiraceae
Intestinimonas
Clostridia_UCG-014
Oscillospiraceae
0 10 20 30 40
Relative contribution (%)
Butyryl-CoA forming enzymes
EC 1.3.1.44
Clostridia_vadinBB60_group
Lachnospiraceae
Anaerostipes
0 20 40 60 80
Relative contribution (%)
EC 1.3.8.1
Others (< 1%)
Lachnospiraceae_NK4A136_group
Clostridia_vadinBB60_group
Anaerotruncus
Colidextribacter
Bilophila
Tuzzerella
Incertae_Sedis
Intestinimonas
Lachnospiraceae
Oscillibacter
Anaerostipes
Oscillospiraceae
ASF356
Clostridia_UCG-014
0 10 20 30 40
Relative contribution (%)
Butyryl-CoA transferase
EC 2.8.3.8
Others (< 1%)
Turicibacter
Clostridia_vadinBB60_group
Clostridia_UCG-014
Oscillibacter
Anaerostipes
Intestinimonas
Oscillospiraceae
0 20 40 60
Relative contribution (%)
P2
EC 2.3.1.9
Others (< 1%)
Turicibacter
Anaeroplasma
GCA-900066575
Anaerotruncus
Clostridia_vadinBB60_group
Colidextribacter
Bilophila
Anaerostipes
Lactobacillus
Incertae_Sedis
Lachnospiraceae_NK4A136_group
Oscillibacter
Intestinimonas
Lachnospiraceae
uncultured
Oscillospiraceae
Clostridia_UCG-014
Lactobacillaceae
0 10 20 30
Relative contribution (%)
EC 1.1.1.35
Rikenellaceae_RC9_gut_group
Turicibacter
Intestinimonas
Oscillospiraceae
uncultured
0 20 40 60 80
Relative contribution (%)
EC 1.1.1.157
Others (< 1%)
Clostridia_vadinBB60_group
Anaerotruncus
[Eubacterium]_coprostanoligenes_group
Anaerostipes
Colidextribacter
Bilophila
Incertae_Sedis
uncultured
Lachnospiraceae_NK4A136_group
Oscillibacter
Lachnospiraceae
Intestinimonas
Oscillospiraceae
Clostridia_UCG-014
0 10 20 30 40
Relative contribution (%)
EC 4.2.1.17
Others (< 1%)
Anaerotruncus
Clostridia_vadinBB60_group
[Eubacterium]_coprostanoligenes_group
Colidextribacter
Bilophila
Tuzzerella
Lactobacillus
Incertae_Sedis
Anaerostipes
Intestinimonas
Lachnospiraceae_NK4A136_group
ASF356
Lachnospiraceae
Oscillibacter
uncultured
Oscillospiraceae
Clostridia_UCG-014
0 20 40 60
Relative contribution (%)

Figure S5. Predicted bacterial contributions to gut microbial butyrate production pathways, related to Figure 5.

Butyrate production pathways were categorized based on the framework described by Vital *et al*.[69] Enzyme Commission (EC) numbers corresponding to butyrate production pathways were identified from the KEGG butanoate metabolism pathway (ko00650). Using 16S rRNA gene sequencing data from HF/HS + acarbose, PICRUSt2 contribution analysis was performed to estimate the contribution of individual bacterial genera to each EC. The relative contribution of each bacterial genus is expressed as a percentage of the total predicted contribution to the respective EC. Data are presented as mean ± SEM (*n* = 8).

Table S2. Nutritional composition of experimental diets, related to Figure 5.

**Product**	D12451	D22122002	D12451px13	Modified D12451px13
	gram (kcal%)	gram (kcal%)	gram (kcal%)	gram (kcal%)
Protein	24 (20)	24 (20)	30 (24)	24 (20)
Carbohydrate	41 (35)	41 (35)	27 (22)	41 (35)
Fat	24 (45)	24 (45)	30 (54)	24 (45)
Total	89 (100)	89 (100)	87 (100)	89 (100)
**Ingredient**	gram (kcal)	gram (kcal)	gram (kcal)	gram (kcal)
Casein	200 (800)	200 (800)	200 (800)	200 (800)
L-Cystein	3 (12)	3 (12)	3 (12)	3 (12)
Corn Starch	72.8 (291)	72.8 (291)	72.8 (291)	72.8 (291)
Maltodextrin 10	100 (400)	100 (400)	100 (400)	100 (400)
Maltose	0	172.8 (691)	0	0
Sucrose	172.8 (691)	0	0	0
Glucose	0	0	0	86.4 (345.5)
Fructose	0	0	0	86.4 (345.5)
Cellulose	50 (0)	50 (0)	50 (0)	50 (0)
Soybean Oil	25 (225)	25 (225)	25 (225)	25 (225)
Lard	177.5 (1598)	177.5 (1598)	177.5 (1598)	177.5 (1598)
Mineral Mix S10026 (Sucrose Based)	10 (0)	0	10 (0)	10 (0)
Mineral Mix S10035 (Dextrose Based)	0	10 (0)	0	0
DiCalucium Phosphate	13 (0)	13 (0)	13 (0)	13 (0)
Calcium Carbonate	5.5 (0)	5.5 (0)	5.5 (0)	5.5 (0)
Potassium Citrate, 1 H_2O	16.5 (0)	16.5 (0)	16.5 (0)	16.5 (0)
Vitamin Mix V10001 (Sucrose Based)	10 (40)	0	10 (40)	10 (40)
Vitamin Mix V10038 (Dextrose Based)	0	10 (40)	0	0
Choline Bitartrate	2 (0)	2 (0)	2 (0)	2 (0)
FD&C Yellow Dye #5	0	0.05 (0)	0	0
FD&C Red Dye #40	0.05 (0)	0	0	0
**Total**	858.15 (4057)	858.15 (4057)	685 (3366)	858.1 (4057)

Table S3. Oligonucleotide sequences used for RT-qPCR in this study, related to Figure 2.

Gene	Primer	Primer sequences
16S	Forward	CACACTGGAACTGAGACACG
	Reverse	TGCTTCTTCTGCGGGTAACG
malE	Forward	AGACACCGATTACTCCATCG
	Reverse	TGGATGGTTGACCCTTGAAG
malF	Forward	GACGAAAATGGCGAGGAACG
	Reverse	AGAGCGGCGTGAAATATCGT
malK	Forward	GCCGAGCCAAGCGTATTTTT
	Reverse	CGTCCAGCACCACGCTTTTG

Table S4. Oligonucleotide sequences used for bacterial quantification in fecal DNA, related to Figure 5.

Target	Primer	Primer sequences	Source
Universal bacterial 16S rDNA	Forward	ACTCCTACGGGAGGCAGCAG	Kuno *et al.*[1]
	Reverse	ATTACCGCGGCTGCTGG	
Bacteroides acidifaciens	Forward	AACCTGATAACGGTGAGCAGGCG	Weiss *et al.*[2]
	Reverse	GCTGACAGCCGAGGTCAATTTG	
Bacteroides ovatus	Forward	CAGGCTCAAGGAAGCACAAG	Kollarcikova *et al.*[3]
	Reverse	CAAAGAACAGCAGGCCATGT	
Bacteroides sartorii	Forward	ATCCAACCTGCCTTATACTCCC	Becattini *et al.*[4]
	Reverse	TACCGAAGTTCTTTAATCACGAGA	
Bacteroides caecimuris	Forward	CGATAGGATATCAAGGTCGCA	This paper
	Reverse	ACCCCTATCCATCGTCGTCT	
Akkermansia muciniphila	Forward	TACAGCCCCGTTCACTCTCAT	Yan *et al.*[5]
	Reverse	TTAACGCTGACGGAGAATTGC	
Parasutterella excrementihominis	Forward	GGAAGTACGGTCGCAAGA	Chen et al.[6]
	Reverse	TGTCAAGGGTAGGTAAGGTTT	

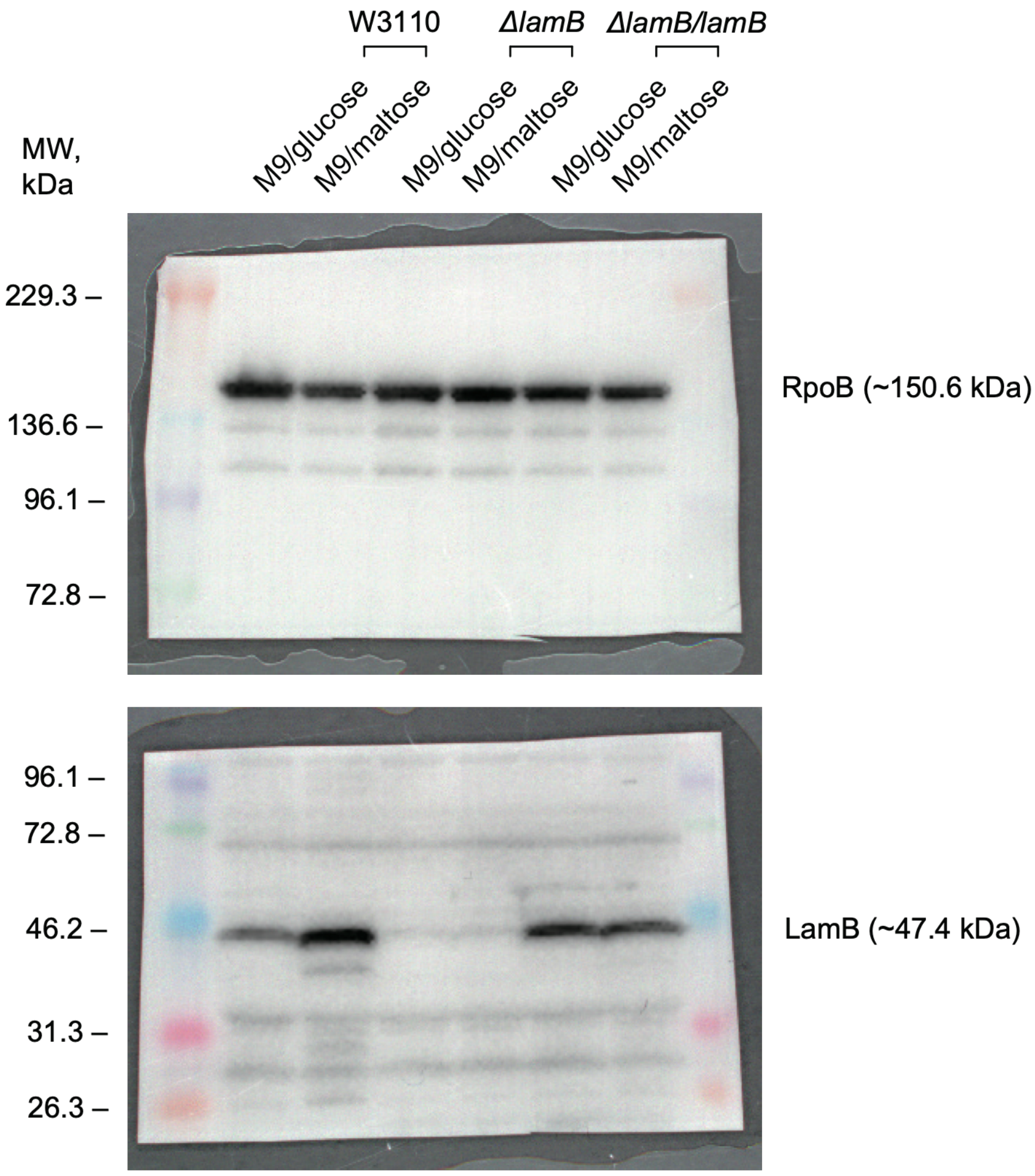

Data S1. Original, Uncropped Western Blot Images

The western blot was performed to confirm the loss of LamB protein in the *E. coli ΔlamB* strain. The images show the complete, original, uncropped membranes, including the molecular weight standards. LamB (~47.4 kDa) was detected as the target protein, with RpoB (~150.6 kDa) used as the loading control. Samples were analysed in one independent experiment (*n* = 1). The blot shown in Figure 2c was obtained from this experiment.

Supplemental references

1. Kuno T, Hirayama-Kurogi M, Ito S, Ohtsuki S. Reduction in hepatic secondary bile acids caused by short-term antibiotic-induced dysbiosis decreases mouse serum glucose and triglyceride levels. Sci Rep. 2018;8(1):1253. Published 2018 Jan 19. doi:10.1038/s41598-018-19545-1
2. Weiss GA, Grabinger T, Glaus Garzon J, et al. Intestinal inflammation alters mucosal carbohydrate foraging and monosaccharide incorporation into microbial glycans. Cell Microbiol. 2021;23(1):e13269. doi:10.1111/cmi.13269
3. Kollarcikova M, Faldynova M, Matiasovicova J, et al. Different Bacteroides Species Colonise Human and Chicken Intestinal Tract. Microorganisms. 2020;8(10):1483. Published 2020 Sep 27. doi:10.3390/microorganisms8101483
4. Becattini S, Sorbara MT, Kim SG, et al. Rapid transcriptional and metabolic adaptation of intestinal microbes to host immune activation. Cell Host Microbe. 2021;29(3):378-393.e5. doi:10.1016/j.chom.2021.01.003
5. Yan Z, Hao T, Yan Y, et al. Quantitative and dynamic profiling of human gut core microbiota by real-time PCR. Appl Microbiol Biotechnol. 2024;108(1):396. Published 2024 Jun 26. doi:10.1007/s00253-024-13204-4
6. Chen YJ, Wu H, Wu SD, et al. Parasutterella, in association with irritable bowel syndrome and intestinal chronic inflammation. J Gastroenterol Hepatol. 2018;33(11):1844-1852. doi:10.1111/jgh.14281
